# 4′-Phosphopantetheinyl Transferase PptT, a New Drug Target Required for *Mycobacterium tuberculosis* Growth and Persistence *In Vivo*

**DOI:** 10.1371/journal.ppat.1003097

**Published:** 2012-12-20

**Authors:** Cécile Leblanc, Thomas Prudhomme, Guillaume Tabouret, Aurélie Ray, Sophie Burbaud, Stéphanie Cabantous, Lionel Mourey, Christophe Guilhot, Christian Chalut

**Affiliations:** 1 CNRS, IPBS (Institut de Pharmacologie et de Biologie Structurale), Toulouse, France; 2 Université de Toulouse, UPS, IPBS, Toulouse, France; 3 Centre de Recherche en Cancérologie (CRCT), UMR 1037 INSERM-CNRS-UPS Institut Claudius Regaud, Toulouse, France; Weill Medical College of Cornell University, United States of America

## Abstract

The cell envelope of *Mycobacterium tuberculosis*, the causative agent of tuberculosis in humans, contains lipids with unusual structures. These lipids play a key role in both virulence and resistance to the various hostile environments encountered by the bacteria during infection. They are synthesized by complex enzymatic systems, including type-I polyketide synthases and type-I and -II fatty acid synthases, which require a post-translational modification to become active. This modification consists of the covalent attachment of the 4′-phosphopantetheine moiety of Coenzyme A catalyzed by phosphopantetheinyl transferases (PPTases). PptT, one of the two PPTases produced by mycobacteria, is involved in post-translational modification of various type-I polyketide synthases required for the formation of both mycolic acids and lipid virulence factors in mycobacteria. Here we identify PptT as a new target for anti-tuberculosis drugs; we address all the critical issues of target validation to demonstrate that PptT can be used to search for new drugs. We confirm that PptT is essential for the growth of *M. bovis* BCG *in vitro* and show that it is required for persistence of *M. bovis* BCG in both infected macrophages and immunodeficient mice. We generated a conditional expression mutant of *M. tuberculosis*, in which the expression of the *pptT* gene is tightly regulated by tetracycline derivatives. We used this construct to demonstrate that PptT is required for the replication and survival of the tubercle bacillus during the acute and chronic phases of infection in mice. Finally, we developed a robust and miniaturized assay based on scintillation proximity assay technology to search for inhibitors of PPTases, and especially of PptT, by high-throughput screening. Our various findings indicate that PptT meets the key criteria for being a therapeutic target for the treatment of mycobacterial infections.

## Author Summary

*Mycobacterium tuberculosis*, the causative agent of human tuberculosis, is responsible for more than 8 million new cases and 1.5 million deaths every year. Despite the existence of effective treatments, the emergence of resistance makes the need for new anti-tuberculosis drugs urgent. The cell envelope of the tubercle bacillus undoubtedly plays a key role in pathogenicity. The envelope has very high lipid content and contains lipids with unusual structures. Some of these lipids are synthesized by complex enzymatic systems that can only become functional after post-translational modification by a 4′-phosphopantetheinyl transferase named PptT. We report that PptT is essential for the viability of *M. tuberculosis in vitro* and of *M. tuberculosis* and its close relative *M. bovis* BCG in both macrophages and the mouse model. Our findings demonstrate that PptT plays a key role in multiplication and persistence of the tubercle bacillus and is therefore an attractive target for drug discovery. We also developed an *in vitro* assay that promises to be a powerful tool for high-throughput screening of PptT inhibitors.

## Introduction

The standard therapy for the treatment of tuberculosis, a disease still responsible for more than 1.5 million deaths and 8 million new cases per year, includes several antibiotics that must be taken for several months (http://www.who.int/tb/dots/treatment). Long-term use of these drugs can cause serious side-effects especially in patients with immunodeficiency disorders and favors the emergence of multidrug-resistant (MDR) and extensively drug-resistant (XDR) mutants which are now starting to pose a serious public health problem [1]. Moreover *M. tuberculosis*, the causative agent of human tuberculosis, is an extraordinarily successful pathogen, able to persist in its human host even when confronted with an intact immune response [2]–[4] and most anti-tuberculosis drugs efficiently kill actively growing tuberculosis bacilli but are less effective against slow replicating or non-replicating bacilli [5], [6]. Therefore, there is an urgent need to improve existing treatment for tuberculosis and, in particular, to find new drugs with the aim of shortening the actual treatment scheme and of providing active molecules to fight MDR and XDR strains.

**Figure 1:**
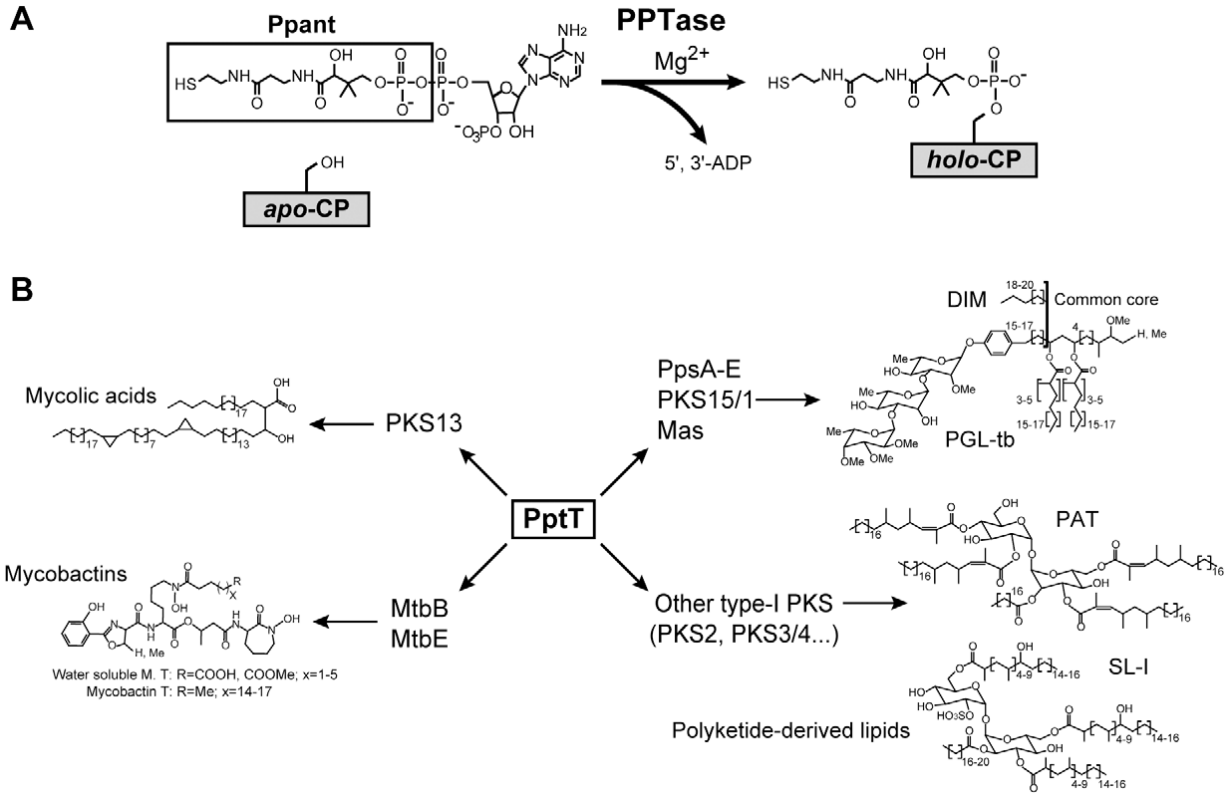
Role of PptT in *M. tuberculosis*. **A.** Enzymatic reaction catalyzed by PPTases. CP: carrier protein. **B.** Schematic diagram of the role of PptT in the biosynthesis pathways for mycolic acids, polyketide-derived lipids and siderophores in *M. tuberculosis*. DIM: phthiocerol dimycocerosates, PGL: phenolglycolipids, PAT: polyacyltrehaloses, SL: sulfolipids.

*M. tuberculosis* has a highly lipid-rich hydrophobic cell wall with a low permeability that contributes to its intrinsic drug resistance [7], [8]. This envelope contains lipids with unusual structures, including mycolic acids which are very long-chain fatty acids found in all mycobacteria, and a number of extractable lipids containing methyl-branched fatty acids that contribute to pathogenicity [9]–[12]. The synthesis of most of these lipids involves large multifunctional enzymes named polyketide synthases (PKS) and two fatty acid synthase (FAS) systems [9], [13]. These enzymes are converted from inactive *apo*-forms to the functional *holo*-forms by the covalent attachment of a 4′-phosphopantetheine (P-pant) group [14], [15]. This reaction is catalyzed by a phosphopantetheinyl transferase (PPTase) which transfers the P-pant group from Coenzyme A (CoA) to the Carrier Protein (CP) domain of the protein (Figure 1A). We have already described two genes in mycobacteria encoding highly conserved PPTases, named AcpS and PptT, and provided direct evidence that these enzymes activate a defined subset of protein substrates in mycobacteria; AcpS is involved in posttranslational modification of FAS-I and PptT modifies the various type-I PKS required for the formation of mycolic acids and lipid virulence factors in *M. tuberculosis* [16]. PptT also activates MbtB and MbtE, two non-ribosomal peptide synthetases (NRPS) involved in the assembly of mycobactin required for *M. tuberculosis* virulence [17]. Thus, PptT plays a major role in the biology of *M. tuberculosis* and related pathogenic mycobacteria, being required for the synthesis of components that are needed for growth and others involved in virulence (Figure 1B). PptT is therefore a potential target for drug development. To test whether PptT is essential for the viability of strains of the *tuberculosis* complex, we generated a conditional *pptT* knockout mutant in *M. bovis* BCG, using a TetR-controlled gene expression system [16], [18]. We found that the expression of *pptT* was required to sustain *M. bovis* BCG growth *in vitro*. Many genes and proteins are required for growth in particular environments [19], but they do not all have the same potential as drug targets. A good drug target enzyme needs to be required for survival in the host environments in particular during chronic infection or for virulence. In addition the enzyme must bind drug-like molecules with high affinity, a feature defined as druggability, and must be vulnerable to inhibition by low doses of small molecule inhibitors. Finally, a simple and robust *in vitro* assay amenable to high-throughput screening is an asset that facilitates the search for potential inhibitors and their improvement. In this study, we addressed these various points for PptT and demonstrate that it fulfills all the requirements for a clinically relevant drug target.

## Results

### *In vitro* growth and biochemical characterization of conditional *pptT* mutants of *M. bovis* BCG and *M. tuberculosis*

**Figure 2:**
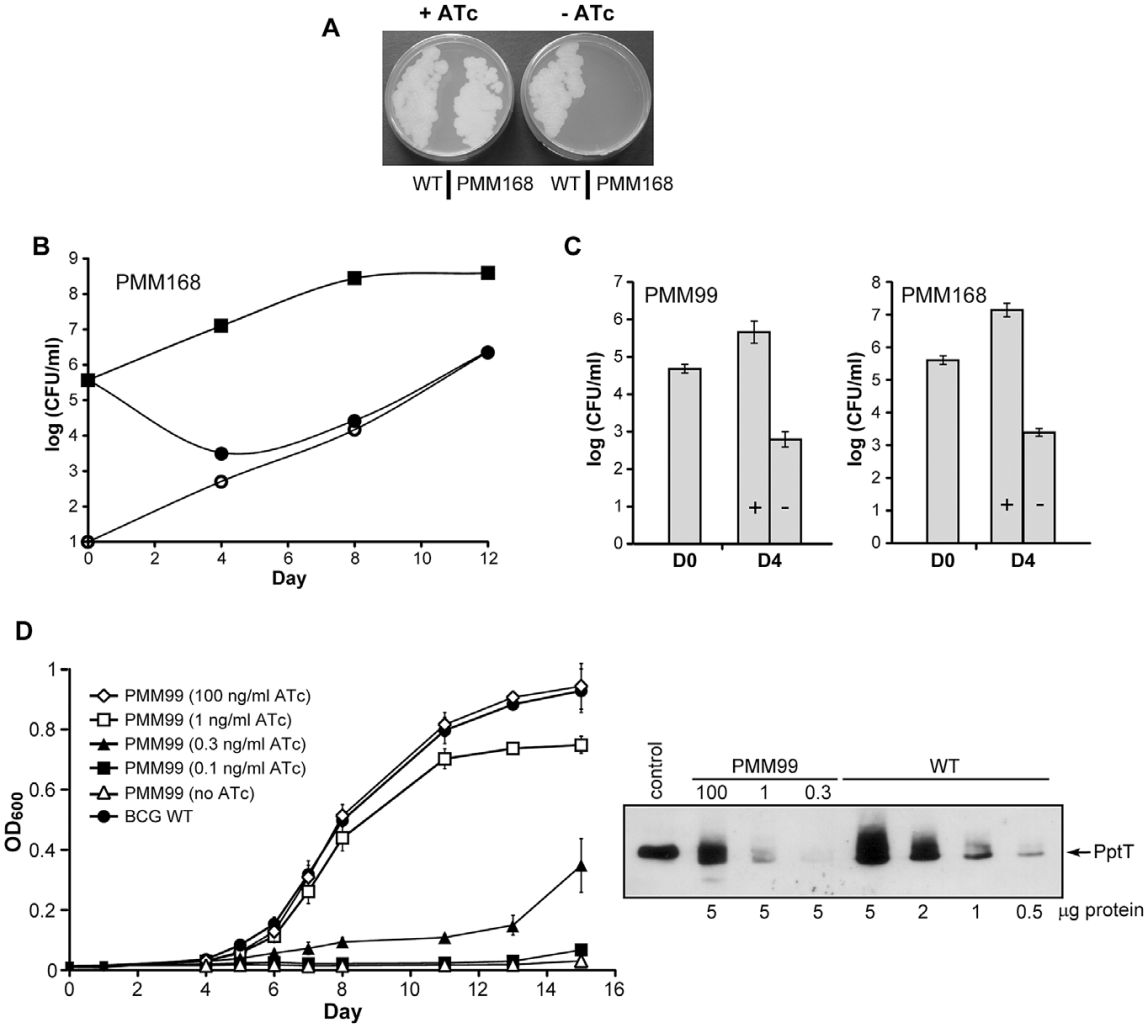
Effect of PptT depletion on the growth of *M. bovis* BCG and of *M. tuberculosis in vitro.* **A.** *M. tuberculosis* H37Rv wild-type (WT) and the PMM168 mutant strain were grown in 7H9 containing ADC (with Km, Hyg and ATc for PMM168) at 37°C and streaked onto 7H11 plates supplemented with OADC with or without ATc (300 ng/ml). Plates were incubated for 20 days at 37°C. **B.** The *M. tuberculosis* PMM168 mutant was grown in 7H9 containing or not containing ATc at 37°C. Numbers of CFU in cultures with ATc (squares) were determined by plating dilutions of theses cultures onto 7H11 plates supplemented with ATc on days 0, 4, 8 and 12. CFU counts in cultures lacking ATc were determined by plating dilutions on 7H11 plates supplemented with ATc (closed circles) or without ATc (open circles) to estimate the number of ATc-independent CFU. **C.** Bactericidal effect of PptT depletion. The *M. bovis* BCG PMM99 (left panel) and *M. tuberculosis* PMM168 (right panel) mutants were grown in 7H9 containing (+) or not containing (−) ATc at 37°C. Numbers of CFU in cultures were determined by plating dilutions of theses cultures onto 7H11 plates supplemented with ATc on days 0 (D0) and 4 (D4). Values are means ± standard deviations (error bars) of CFU counts for three independent experiments. **D.** PMM99 was grown in media supplemented with Tween-80 with (100, 1, 0.3, 0.1 ng/ml) and without ATc and bacterial growth was monitored by measuring the optical density at 600 nm (OD_600_) (left panel). *M. bovis* BCG wild-type strain was grown in 7H9 supplemented with Tween-80. Data are representative of two independent experiments. Western blot visualization of PptT in crude cell lysates of PMM99 (5 µg/lane) cultivated for 6 days in a medium with ATc (100, 1, 0.3 ng/ml) and in a crude cell lysate of *M. bovis* BCG wild-type strain (5, 2, 1, 0.5 µg/lane) (right panel). The control lane was loaded with 100 ng of recombinant PptT fused to a poly-histidine tag produced in *E. coli*.

We previously described the construction of a conditional *pptT* expression mutant of *M. bovis* BCG, named PMM99, based on the use of a TetR/*tetO* expression regulation system [16]. We generated a similar mutant, named PMM168, in *M. tuberculosis* H37Rv using the same strategy as for the construction of PMM99 (Figure S1 and [16]). Both mutants grew normally on 7H11 plates supplemented with anhydrotetracycline (ATc; 100 ng/ml for the *M. bovis* BCG mutant and 300 ng/ml for the *M. tuberculosis* mutant) but were unable to grow on plates in the absence of ATc, in contrast to the wild-type strain (Figure 2A and [16]) indicating that expression of *pptT* is required for growth on solid medium. Note that the concentration of ATc required for the *M. tuberculosis* mutant was higher than for the *M. bovis* BCG mutant.

To confirm that PptT is required for the growth of *M. tuberculosis* complex strains *in vitro*, strains PMM99 and PMM168 were cultivated in 7H9 with or without ATc (100 ng/ml) and bacterial growth was evaluated by plating serial dilutions of each culture on solid medium after various times of culture. The mutant strains exhibited a typical exponential growth curve when grown in the presence of ATc but their growth was inhibited in the absence of ATc (Figure 2B for PMM168 and data not shown for PMM99). Surprisingly, growth was restored in the ATc-free medium from day 6 suggesting that a population of ATc-independent mutants emerged and multiplied in the cultures. This was confirmed by plating dilutions of *M. tuberculosis* ATc-free culture on solid medium with and without ATc. The ATc-independent colony forming unit (CFU) counts were 4.57 log lower than the ATc-dependent CFU counts in the initial population but increased during the course of the experiment to become the main population after 8 days (Figure 2B). ATc-independent clones were isolated and the *tetR* gene sequenced: mutations affecting the expression or the amino-acid sequence of the repressor were detected (data not shown). The same behavior was observed with the PMM99 mutant (data not shown). These results strongly support our hypothesis that TetR repression was abolished in these clones.

This initial analysis revealed that PptT depletion due to the absence of ATc in the growth medium, is associated with death of bacteria. This was confirmed by comparing in multiple independent cultures the number of CFU after 4 days of culture in medium with and without ATc. We found that the mean number of CFU in *M. bovis* BCG and *M. tuberculosis* cultures without ATc were 2.87 and 3.75 log, respectively, lower than in cultures with ATc indicating that PptT is required for the growth of both strains in liquid medium (Figure 2C). Moreover CFU counts in *M. bovis* BCG and *M. tuberculosis* cultures without ATc were drastically (2 log) reduced compared to the CFU counts in the initial population indicating that PptT depletion is bactericidal for both strains.

We next assessed the control of *pptT* expression in the recombinant *M. bovis* BCG strain. The PMM99 strain was cultivated in liquid media with different ATc concentrations and cell growth was followed for 15 days by optical density measurements at 600 nm (OD_600_). Growth of PMM99 was indistinguishable from that of wild-type *M. bovis* BCG when ATc was used at concentrations ranging from 100 to 2 ng/ml. A clear growth defect was observed at concentration below 1 ng/ml ATc with no detectable growth in the presence of 0.1 ng/ml ATc and in ATc-free medium (Figure 2D).

To correlate cell growth and expression of PptT, total cell lysates of PMM99 grown for 6 days in the presence of 100, 1 or 0.3 ng/ml ATc were studied by western blotting with an anti-PptT antibody. The amount of PptT in the PMM99 strain exposed to 100 ng/ml ATc was approximately 2.5 times lower than the amount of PptT in the wild-type strain (Figure 2D). When compared to the wild-type, PptT production was decreased by ∼10-fold and 20-fold in the PMM99 strain exposed to 1 and 0.3 ng/ml ATc indicating that activity of PptT should be reduced by at least 95% to inhibit the growth of *M. bovis* BCG *in vitro*. Coomassie blue staining after SDS-PAGE and western blotting with an anti-Hsp65 antibody indicated that there were similar amounts of total protein in the cell extracts (Figure S2). These experiments show that the expression of the *pptT* gene in PMM99 was tightly regulated by ATc and that the transcriptional shutdown of the *pptT* gene in the mycobacterial cell correlated with a severe growth defect.

### PptT is required for persistence of mycobacteria in infected macrophages

**Figure 3:**
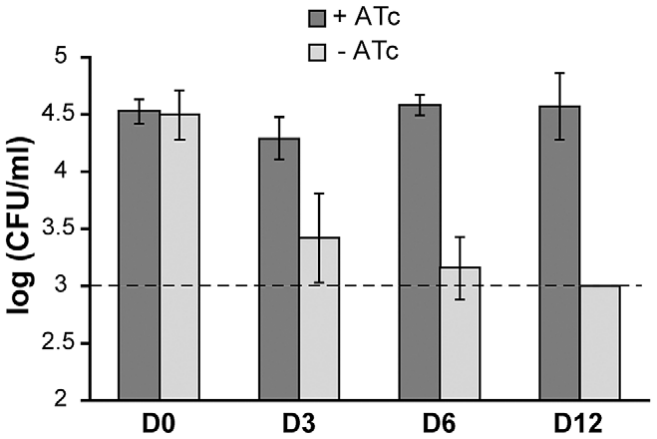
Effect of PptT depletion on survival of PMM99 in murine macrophages. Bone marrow-derived macrophages were infected with strain PMM99, cultivated in the presence or absence of ATc (500 ng/ml) and lysed after 0, 3, 6 or 12 days. Viable bacteria were counted by plating dilutions of the lysates on selective media. The data reported are from one experiment performed in triplicate and representative of three independent experiments. The dashed line corresponds to the detection limit (3 log, see materials and methods).

We examined whether PptT is required for the intracellular survival of mycobacteria in macrophages, the primary cellular targets for *M. tuberculosis*. This included testing whether the TetR-controlled expression system could regulate the expression of *pptT* in intracellular bacteria. Murine bone-marrow derived macrophages were infected with PMM99 and cultivated in a medium either containing (500 ng/ml) or not containing ATc. Infected macrophages were lysed 3, 6 or 12 days post-infection and the numbers of viable bacteria counted. The number of CFU in ATc-treated macrophages remained stable throughout the experiment (around 3.7×10^4^ CFU/ml, Figure 3), whereas the bacterial load decreased substantially in macrophages cultivated in the absence of ATc. On day 12, there were 1.5 log fewer CFU in macrophages cultivated without than with ATc (Figure 3). Thus, the expression of *pptT* was controlled by the TetR system in macrophages, and was required to maintain mycobacterial viability.

### PptT is essential for the multiplication of mycobacteria in mice

**Figure 4:**
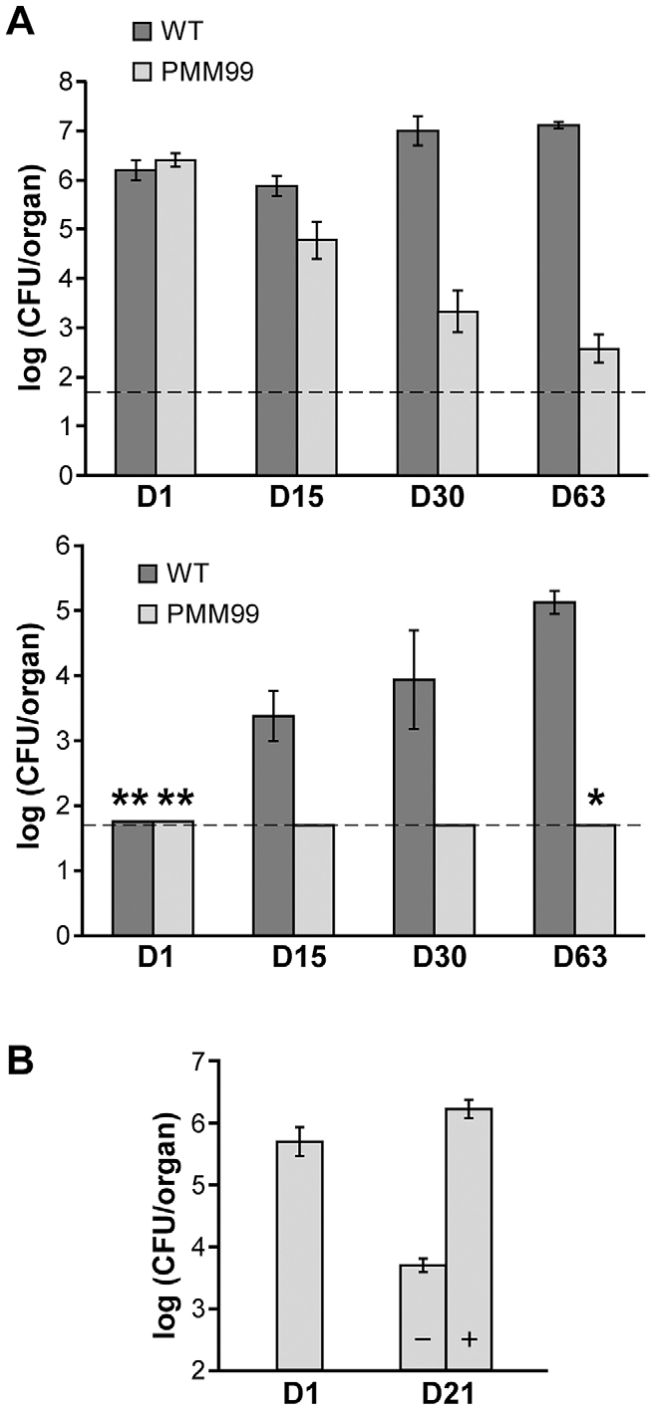
SCID mice infections with the PMM99 mutant. **A.** Competition between strain PMM99 and wild-type *M. bovis* BCG in infected SCID mice. SCID mice were infected with a mixture of wild-type *M. bovis* BCG and PMM99. Numbers of CFU of each strain recovered from the lungs (upper panel) and the spleen (lower panel) of SCID mice 1 day (D1), 15 days (D15), 30 days (D30) and 63 days (D63) after infection were determined by plating dilutions of homogenized tissues on selective media. Values are means ± standard deviations (error bars) of CFU counts for 4 (day 63) or 5 (days 1, 15, 30) infected mice. The dashed lines correspond to the detection limit (1.7 log, see materials and methods). Asterisks indicate that CFU counts in all (*) or some (**) infected mice were below the detection limit (50 CFU/organ): the number of CFU scored in such cases is 50 CFU per organ. The average number of CFU recovered from the organ is therefore overestimated. **B.** Effect of doxycycline treatment on the survival of PMM99 in SCID mice. SCID mice were infected with strain PMM99 and administered or not administered 0.1 mg/ml doxycycline. Numbers of CFU recovered from the lungs of infected mice on day 1 (D1) and from the lungs of untreated (−) and treated (+) mice on day 21 (D21) were determined by plating dilutions of homogenized tissues on 7H11 media. Values are means ± standard deviations (error bars) of CFU counts for three infected mice.

Next, we sought to establish whether PptT contributes to mycobacterial fitness during infection of mice. We first used mice with severe combined immune deficiency (SCID), an environment more complex than that of *ex vivo* macrophages but less complex than immunocompetent hosts due to the lack of adaptive immune response. We used the *M. bovis* BCG conditional mutant PMM99 such that the infected mice would not die rapidly and thereby allow the infection to be monitored for a substantial period. A competitive mixed-infection assay was carried out with PMM99 and a *M. bovis* BCG wild-type strain harboring a streptomycin resistance gene (*M. bovis* BCG:pMV361st). SCID mice were infected with an infectious dose of 5×10^6^ CFU of each strain (a 1/1 ratio). The mice were killed 1, 15, 30 or 63 days post-infection, and the mutant and wild-type loads in lungs and spleen were determined by plating on selective medium. CFU counts in lungs remained almost stable for 63 days for the wild-type strain but declined massively for strain PMM99 (Figure 4A): 63 days post-infection an average of 1.29×10^7^ CFU/organ of the wild-type strain were recovered but only 3.71×10^2^ CFU/organ of strain PMM99 such that the ratio of PMM99 to wild-type bacteria fell by more than 30000-fold in lungs between day 0 and day 63. The numbers of CFU in spleens remained below the threshold of detection (1.7 log) throughout the experiment for strain PMM99 and increased progressively for the wild-type strain to reach a mean of 1.3×10^5^ CFU/organ by day 63 (Figure 4A).

To confirm that the reduced survival of the PMM99 mutant in SCID mice was indeed related to *pptT* silencing, we examined the ability of the mutant strain to survive in SCID mice treated with doxycycline, a tetracycline derivative used to switch on the TetR regulator system in infected mice [20], [21]. However, continuous administration of substantial doses of doxycycline may be toxic for the bacteria. Therefore, we determined the highest concentration of doxycycline that could be used without affecting the survival of *M. bovis* BCG as follows. SCID mice were infected with the wild-type *M. bovis* BCG strain (infectious dose of 4×10^5^ CFU), and the bacterial load in lungs of three mice was determined the next day. The remaining mice were treated for 21 days with doxycycline (provided at 0, 0.1, 0.3 or 1 mg/ml in drinking water). There was no significant difference between CFU counts in the lungs of mice treated with 0.1 mg/ml of doxycycline and untreated controls (Figure S3); the CFU counts on day 21 were, by contrast, lower in mice treated with higher doxycycline concentrations. Therefore, doxycycline concentrations of 0.3 mg/ml and above in drinking water interfered with the survival of *M. bovis* BCG in SCID mice. Based on these data, SCID mice were infected with the strain PMM99 (4.79×10^5^ CFU per mouse). Three mice were sacrificed the next day to evaluate bacterial uptake in lungs, and the other mice were given drinking water with or without 0.1 mg/ml doxycycline. Twenty-one days post-infection, there was a marked difference in CFU counts between treated and untreated mice. In mice receiving doxycycline, CFU counts in lungs were similar to those in the lungs of mice killed 1 day post-infection, consistent with our initial competition experiment in which the BCG load was stable for at least 63 days. In contrast, the CFU counts in lungs of untreated mice were about 2 log lower on day 21 (Figure 4B). This experiment confirms that survival of PMM99 in mice was dependent on the production of PptT, and indicates that a doxycycline concentration of 0.1 mg/ml in drinking water induced the expression of the *pptT* gene at a level sufficient to sustain the infection.

**Figure 5:**
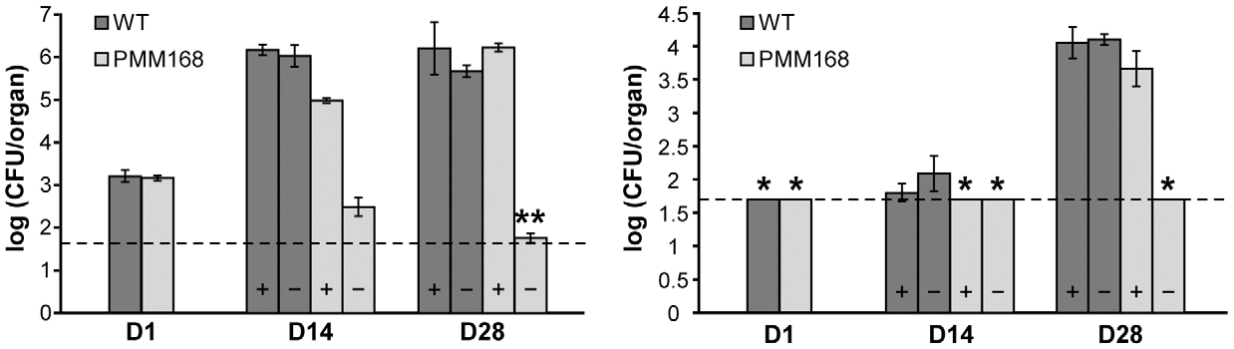
Effect of PptT depletion on the multiplication of *M. tuberculosis* H37Rv in BALB/c mice. Mice were infected either with the wild-type *M. tuberculosis* strain or with strain PMM168 and either received or did not receive doxycycline treatment (0.1 mg/ml) from one day post-infection. Numbers of CFU of *M. tuberculosis* wild-type (dark gray bars) or of PMM168 (light gray bars) in lungs (left panel) and spleen (right panel) of treated (+) and untreated (−) mice were determined on days 1 (D1), 14 (D14) and 28 (D28) by plating dilutions of homogenized tissues on selective media. Values are means ± standard deviations (error bars) of CFU counts for three or four infected mice (see materials and methods). The dashed line corresponds to the detection limit (1.7 log). Asterisks indicate the counts in all (*) or some (**) infected mice were below the detection limit (50 CFU/organ). In such cases, the number of CFU scored was 50 CFU per organ; consequently mean number of CFU per organ is an overestimation.

Next, we increased the complexity of the environment encountered by the *pptT* conditional mutant by using immunocompetent mice. We used the conditional *pptT* mutant of *M. tuberculosis* because *M. bovis* BCG is eliminated from immunocompetent mice. BALB/c mice were infected with similar-sized inocula of either strain PMM168 or the *M. tuberculosis* H37Rv wild-type strain, and provided with drinking water containing or not containing 0.1 mg/ml doxycycline. Mice were killed 1, 14 or 28 days post-infection and the numbers of bacteria present in lungs and spleen were determined. The counts of the wild-type strain in each spleen and lungs did not differ between untreated and doxycycline-treated mice indicating that the concentration of doxycycline used did not affect its viability or spread (Figure 5). After 28 days of infection, the counts in lungs of treated mice were 1.70×10^6^ CFU/organ for PMM168 and 1.62×10^6^ CFU/organ for the wild-type strain. The counts in spleens were similar for the two strains (4.57×10^3^ CFU/organ and 1.12×10^4^ CFU/organ). Thus, *pptT* expression induced by treating infected mice with doxycycline (0.1 mg/ml in drinking water) was sufficient to allow growth *in vivo* similar to that of the wild-type. However, on day 14, there was a reproducible slight difference in CFU counts between treated mice infected with wild-type and those infected with PMM168 suggesting that *pptT* expression and polyketides production in the mutant were not exactly the same as those in the wild-type strain. The difference between wild-type and mutant was much larger in the absence of doxycycline, with the mutant being much less abundant in both lungs and spleen: the counts for lung were 3.09×10^2^ CFU/organ after 14 days and 5.80×10^1^ CFU/organ after 28 days. Thus, after 28 days of infection there were at least 4.5 log fewer CFU in the lungs of untreated than treated mice. The mutant was undetectable in the spleen of untreated mice throughout the experiment. These experiments demonstrate that mycobacterial multiplication in both immunodeficient and immunocompetent mice is dependent on the expression of PptT.

### PptT is essential for the persistence of *M. tuberculosis* in mice

**Figure 6:**
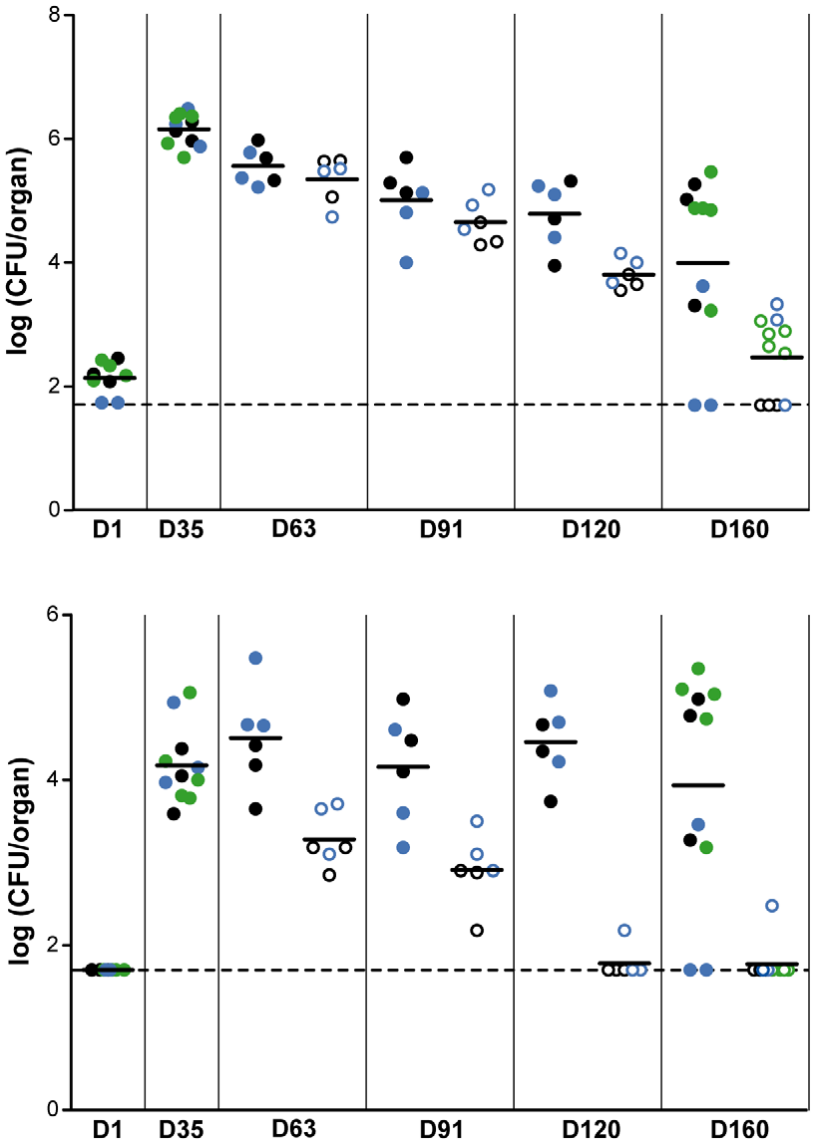
Effect of PptT depletion on the persistence of *M. tuberculosis* H37Rv in BALB/c mice. Mice were infected with strain PMM168 and treated with 0.1 mg/ml doxycycline from 1 day post-infection. Thirty-five days post-infection mice were split in two groups: group 1 continued to receive doxycycline and doxycycline treatment was withdrawn from group 2. Numbers of CFU present in lungs (upper panel) and spleen (lower panel) of treated (closed circles) and of untreated (open circles) mice were determined 1 (D1), 35 (D35), 63 (D63), 91 (D91), 120 (D120) and 160 (D160) days post-infection (experiments 1 and 2, black and blue circles) or 1, 35 and 160 days post-infection (experiment 3, green circles). Each circle represents CFU obtained from one mouse. The dashed line corresponds to the detection limit. When counts in infected mice were below the detection limit, the number of CFU scored was 50 CFU per organ (1.7 log).

One of the criteria for validating a new drug target is that it should be required for pathogen persistence in the host. It is believed that during human infection, a pool of mycobacteria persists in a slow-replicating state less sensitive to drugs [4]. This slow-replicating state is observed in immunocompetent mice after 4 weeks of infection [22]. To address this issue, mice were infected with the *M. tuberculosis* conditional mutant and maintained on doxycycline (0.1 mg/ml in drinking water) for 35 days to allow establishment of mycobacterial infection, acute phase multiplication and initiation of the immune response. Doxycycline was then withdrawn from half of the mice while the other half continued to receive it. Bacterial loads in lungs and spleens were determined on days 1, 35, 63, 91, 120, 160 post-infection. At 35 days post-infection, the mean bacterial counts were 1.45×10^6^ CFU/organ in lungs and 1.51×10^4^ CFU/organ in spleen, confirming that doxycycline treatment sustained growth of PMM168 in mice (Figure 6). During the chronic phase, we observed a rapid drop in bacterial loads in untreated animals. The mycobacterial load in the lungs of untreated mice fell to below the threshold of detection on day 160 in four of the eleven animals and to 3 log below the value for day 35 in the other animals. The mycobacterial load in the spleen was below the threshold in five of the six mice on day 120 and in ten of the eleven mice on day 160. Surprisingly, the mean lung CFU counts also declined in infected mice treated with doxycycline. We also observed that the CFU counts at day 160 differed substantially between individual animals with bacterial loads in the lungs and spleen ranging from a value close to that at day 35 for 6 mice to less than 50 CFU/organ for 2 mice, the other 3 being in between (Figure 6). This was unexpected because the load of wild-type bacteria remains constant over a period of several months during the chronic phase of infection in mice [23]. In addition, the bacterial burden was very similar between individuals at days 1 and 35 post-infection indicating that each mice received similar inoculum size and that growth during the acute phase was similar and consistent with previous experiments. The large dispersion of bacterial load did not reflect the emergence of a TetR-independent population because no CFU was obtained on ATc-free plates with any mice and at any time points. Therefore, the most likely explanation for the lack of PMM168 persistence in a few mice treated with doxycycline is that PptT production was not high enough, possibly because the doxycycline concentration at the site of mycobacterial persistence in these mice was not high enough. Consistent with this explanation, we observed that the amount of PptT produced in PMM99 *in vitro* is lower than in the wild-type strain, even in the presence of high concentration of inducer (Figure 2D). This reduced production has no impact on growth *in vitro* but could affect survival during the chronic phase, especially if PptT expression is further reduced in the mutant at late stages of infection or if higher amounts of enzyme are required for stable persistence. However, the lower persistence of the PMM168 mutant in both lungs and spleen of doxycycline-untreated mice, clearly indicates that PptT production is required for survival of *M. tuberculosis* during the chronic phase of infection.

### Development of an *in vitro* assay for high-throughput screening of PptT inhibitors

**Figure 7:**
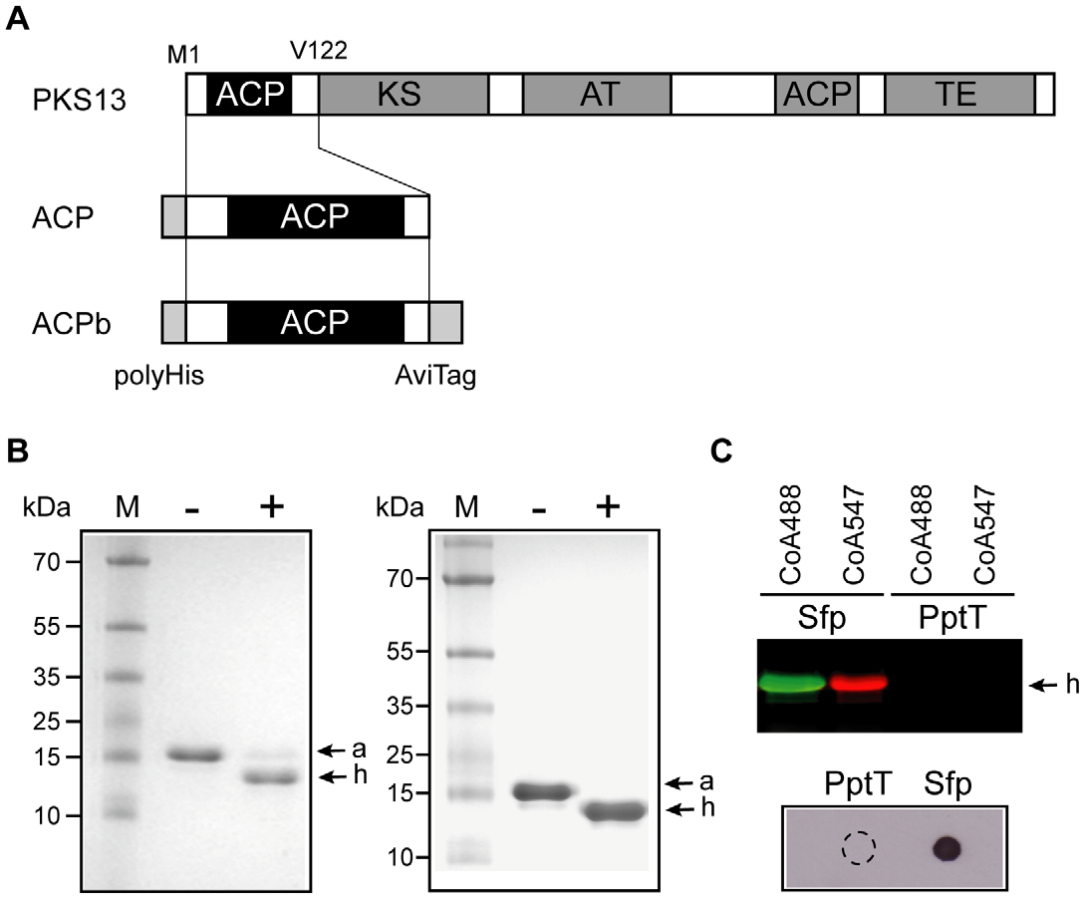
*In vitro* activity of PptT. **A.** Diagrammatic representation of the domain organization of PKS13 and of ACP and ACPb domains. ACP: Acyl Carrier protein, KS: ketosynthase, AT: acyltransferase, TE: thioesterase. **B.** ACP activation with CoA and acetyl-CoA. The *apo*-ACP module was incubated with (+) or without (−) PptT in the presence of either CoA (left panel) or acetyl-CoA (right panel). *apo*- (a) and *holo*-ACP (h) forms were separated on urea polyacrylamide gels and stained with Coomassie blue (see materials and methods). M: PageRuler prestained protein ladder plus (Fermentas). **C.** ACP activation with CoA analogs. PptT or Sfp were incubated with the *apo*-ACP domain in the presence of either fluorescent CoA analogs (CoA488 or CoA547) or CoA-biotin. Fluorescent *holo*-ACP forms (h) were resolved by SDS-PAGE and visualized by fluorescence scanning using a Typhoon scanner (GE Healthcare) (upper panel). Biotin-labeled ACP was detected by spotting 5 µl of the reaction mix onto the nitrocellulose membrane and incubation with streptavidin peroxidase followed by enhanced chemiluminescence detection (lower panel). The dashed-line circle shows the drop zone for the PptT reaction.

As PptT is essential for *M. tuberculosis,* it is a potential target for the development of new anti-tuberculosis drugs. We sought to design an assay for high-throughput screening (HTS) of PptT inhibitors. The PptT enzyme fused to the Maltose Binding Protein (MBP-PptT) was overproduced and purified by amylose affinity chromatography. An ACP module, corresponding to the N-terminal ACP domain of PKS13 (Figure 7A), was overproduced in a *E. coli* strain defective in the production of the endogenous EntD PPTase that could partially activate the ACP domain [16]. The purified MBP-PptT in the presence of CoA and Mg^2+^ converted *apo* to *holo*-ACP (Figure 7B) demonstrating that our MBP-PptT construct was functional *in vitro* and that the ACP module isolated serves as a substrate for PptT. MBP-PptT enzyme displayed a maximal activity at 30°C in the pH range of 7.0 to 8.0 (Figure S4). Under these conditions, we found that PptT efficiently transferred the acetylated P-pant group from acetyl-CoA to the ACP domain (Figure 7B).

HTS of enzyme inhibitors requires simple, efficient and sensitive means for assaying the reaction products. A number of PPTases, including Sfp, a PPTase from *Bacillus subtilis* with broad substrate specificity, can catalyze modification of carrier proteins with CoA analogs harboring a fluorescent or an affinity reporter at the end of the P-pant arm. This provides a sensitive means of visualizing the protein product [24]–[26]. We tested whether this approach could be used with MBP-PptT. MBP-PptT and Sfp were assayed in the presence of fluorescent (CoA488 and CoA547) and biotinylaled CoA derivatives separately. As expected, Sfp efficiently catalyzed the conversion of *apo*-ACP into *holo*-ACP upon incubation with these CoA analogs (Figure 7C). However, no fluorescent or biotinylaled products were detected with PptT indicating that the enzyme was unable to use CoA derivatives bearing thioester substituents as substrate (Figure 7C).

**Figure 8:**
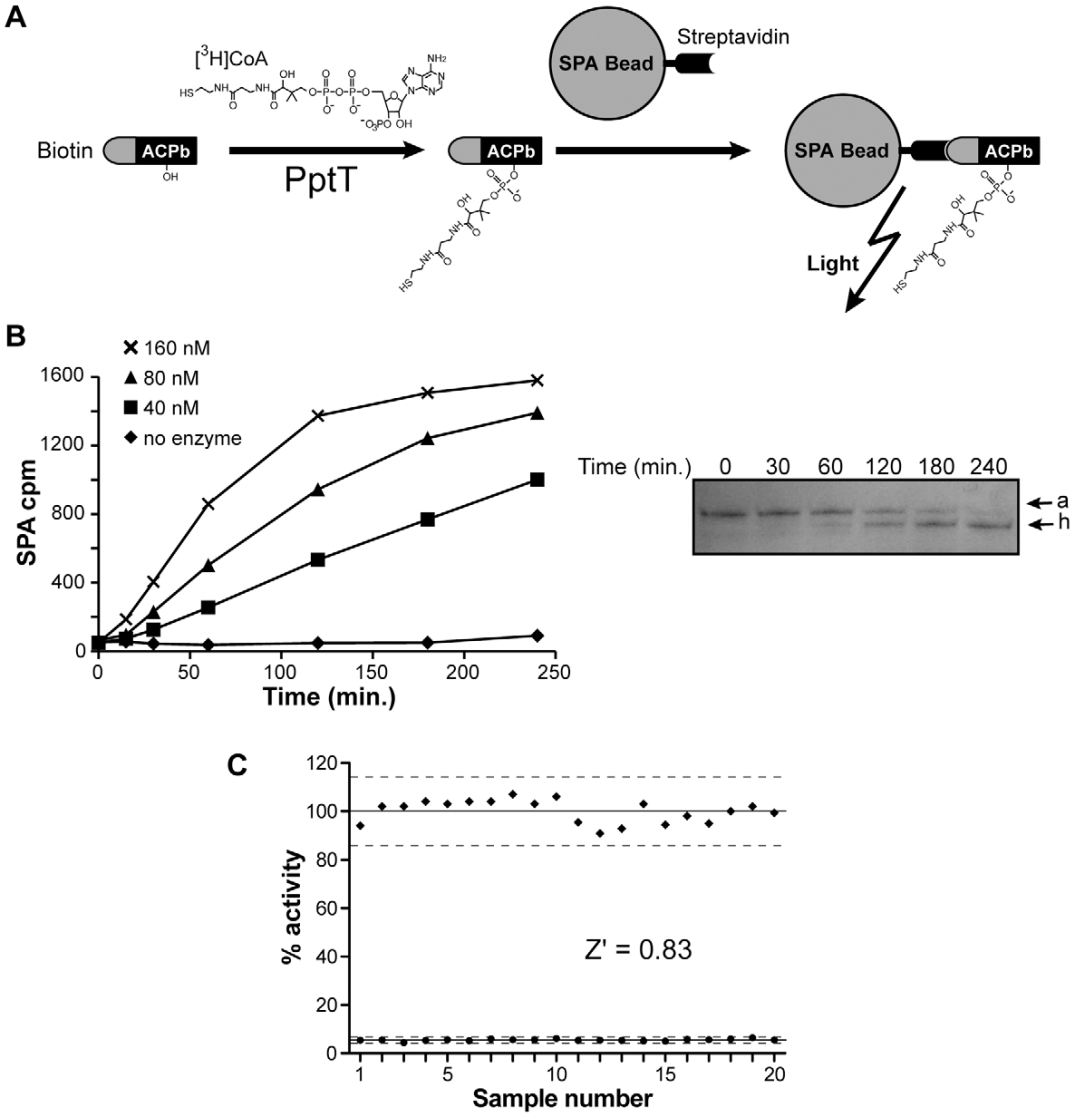
PptT SPA assay for high throughput screening. **A.** Principle of the PptT SPA assay. **B.** Effect of enzyme concentration on the SPA assay signal. Biotinylated *apo*-ACPb (4 µM) and radiolabeled [^3^H]CoA (2 µM) were incubated in the presence of 0, 40, 80, or 160 nM of PptT in standard conditions (see materials and methods). Reactions were stopped after various times by addition of stop buffer and 250 µg of SPA beads resuspended in 60 µl of water. Signals were detected by scintillation counting using a TopCount (Perkin Elmer). A parallel kinetic experiment was carried out with 80 nM of enzyme: the reaction was stopped at various times and loaded onto a urea polyacrylamide gel to separate *apo*- (a) and *holo*-ACP (h) forms (right panel). The gel was stained with Coomassie blue. **C.** Determination of the Z′ factor. SPA assays were carried out in standard conditions (4 µM of ACPb, 2 µM of [^3^H]CoA, 2 µM of CoA) in the presence of 80 nM of enzyme (n = 20, black squares) or without enzyme (n = 20, black circles) at 30°C for 1.5 hour in a 96-well plate. Reactions were stopped by addition of stop buffer and SPA beads, and scintillation signals were detected using a TopCount. The Z′ factor was calculated as indicated in materials and methods (μ_c+_ = 100, μ_c−_ = 5.53, σ_c+_ = 4.74, σ_c−_ = 0.42). The dotted lines correspond to three standard deviations from the mean of the positive and negative controls. Data are expressed relative to the mean value for positive controls.

We therefore exploited the Scintillation Proximity Assay (SPA) technology. The SPA system involves the use of microspheres (SPA beads) which emit light when a radiolabeled molecule is attached or is in proximity [27]. The PptT enzyme was incubated with the ACP module in the presence of [^3^H]CoA to generate [^3^H]*holo*-ACP, the formation of which was monitored by counting scintillation from SPA beads (Figure 8A) [27]. To ensure high binding efficiency of the ACP domain to the beads, we used streptavidin-coated SPA beads and a biotinylated ACP module (ACPb). ACPb was generated by producing the ACP domain fused to a C-terminal biotin acceptor domain (AviTag) and subsequent *in vitro* biotinylation using BirA [28] (Figures 7A and 8A).

Assays were run at 30°C in a final volume of 10 µl in 96-well microplates and reactions were stopped by addition of stop buffer and SPA beads resuspended in water. Assay conditions were optimized as follows: 4 µM (720 ng/assay) of ACPb substrate and 4 µM of CoA with a [^3^H]CoA/CoA ratio of 1/1 (2 µM for each component). Under these conditions, 250 µg of SPA beads per well was sufficient to capture all the input substrate. Lower concentrations of ACPb or [^3^H]CoA resulted in lower output signal intensity; higher concentrations of ACPb or [^3^H]CoA improved the output signal intensity but increased the signal-to-noise ratio (data not shown), and costs (more beads and more radioactive material per assay). We then established the optimal concentration of enzyme by kinetic experiments with concentrations of enzyme of 40 nM to 160 nM. The rate of reaction increased significantly as the concentration of enzyme increased (Figure 8B). An enzyme concentration of 80 nM was chosen for the assay because it was a good compromise between output signal intensity and the use of a large excess of substrate over enzyme concentration (enzyme-to-substrate ratio of 1∶50). To correlate SPA assay signal intensity to the production of [^3^H]*holo*-ACPb, a parallel kinetic experiment was conducted in the same conditions with 80 nM of enzyme; reaction product formation was estimated by loading each reaction mix on a urea-polyacrylamide gel to separate the *apo*- and *holo*-ACPb forms. The signal increased in direct proportion to the amount of *holo-*ACPb produced in the reaction (Figure 8B).

Assay quality was assessed by determining the Z′ factor, a statistical parameter used for evaluation and validation of high-throughput assays [29]. The assay was shown to have a Z′ factor of 0.83: a value greater than 0.5 indicates high quality for an HTS assay (Figure 8C). We also examined the effect of DMSO on the assay signal because this solvent is often used in HTS assays to dissolve test compounds. DMSO concentrations up to 3% had no effect on signal intensity; the presence of 4 and 5% DMSO reduced the signal by 20%. Thus the assay was sufficiently tolerant to DMSO (Figure S5). This new assay is thus a robust and stable assay for monitoring PptT activity, involves a minimum of sample handling and does not require separation steps, making it suitable for HTS applications.

## Discussion

The main goal of this study was to evaluate whether PptT is a new target suitable for the search for new anti-tuberculosis drugs. We addressed the various important criteria allowing target validation. We established that PptT is required for mycobacterial survival in various environments, including those encountered in macrophages, and during the various phases of infection in an animal model. We also developed a robust enzymatic assay in a format compatible with robotic systems for high-throughput screening of large libraries of compounds. The Z′ factor for this assay was 0.83 demonstrating its stability and its potential for selecting PptT inhibitors. Therefore, we established that PptT is a highly promising target for the development of new anti-tuberculosis drugs. A conserved PptT-encoding gene is present in all sequenced mycobacterial species, including clinical isolates, providing further evidence for its essential role and PptT shares only low amino acid conservation (12% identity) with the human PPTase (UniProtKB/Swiss-Prot accession number Q9NRN7), facilitating selective targeting of the mycobacterial protein.

We constructed and analyzed two conditional *pptT* mutants, one in *M. bovis* BCG and one in *M. tuberculosis* H37Rv: the wild-type allele of *pptT* was deleted; *pptT* was placed in a replicative plasmid under the control of TetR-controlled promoter *p_myc1_tetO* (the plasmid also contained *tetR*). The amount of PptT synthesized was dependent on inducer concentration and 95% depletion of PptT was required to inhibit *in vitro* growth of *M. bovis* BCG. Vulnerability of only one protein, but no known target of efficient antibiotics such as RpoB for rifampicin or InhA for isoniazid, has been evaluated in *M. tuberculosis* complex strain using a comparable genetic system. This certainly limits the assessment of the PptT vulnerability in comparison to that of other targets, either proved or proposed. However, it was reported that reducing expression of BioA, an enzyme involved in protein biotinylation, by 99% was required to prevent growth of *M. tuberculosis* [30]. In *Mycobacterium smegmatis*, a 80% depletion of RpoB blocks bacterial multiplication but 97% decrease in DHFR amount, the target of trimethoprim, has little effect on growth *in vitro* [31]. Therefore, the case of PptT seems intermediate.

Interestingly, a higher concentration of ATc was needed in solid medium for growth of the *M. tuberculosis* mutant than that of the *M. bovis* BCG mutant although the constructs were identical. Possibly, production of PptT was differently regulated by the TetR-controlled expression system in the two strains. Alternatively, higher levels of PptT are required for *M. tuberculosis* growth, or the cell envelope of *M. tuberculosis* may be less permeable to ATc than that of *M. bovis* BCG. Our findings do not allow us to eliminate any of these hypotheses. The main purpose of this study was to evaluate whether PptT represents a new target for the development of new anti-tuberculosis drugs by investigating its role in the biology of two *M. tuberculosis* complex strains, *M. bovis* BCG and *M. tuberculosis*. PptT activates the various type-I PKS in mycobacteria, including PKS13, an enzyme essential for mycolic acid biosynthesis; it also activates several NRPS proteins required for assembly of the virulence-conferring siderophore mycobactin [16], [17]. Thus, inhibition of PptT activity may prevent the formation of components essential for mycobacterial viability or involved in pathogenicity.

Although PptT was previously shown to be essential for the *in vitro* growth of *M. bovis* BCG [16], its role in *M. tuberculosis* viability and mycobacterial survival in intracellular environments or animal models had not been investigated. This is important for the potential of PptT as drug target because this enzyme is involved in the biosynthesis of various cell wall components including mycolic acids, polyketides, siderophores and, except for the latter, their contributions to the pathogenesis of *M. tuberculosis*, particularly during the chronic phase of infection, remain elusive. In addition some compensation mechanisms might counteract PptT deficiency in *ex vivo* or *in vivo* environments thereby allowing *M. tuberc*ulosis survival. Consistent with this, some enzymatic functions that are essential *in vitro* in mycobacteria appear to be dispensable *in vivo*. For instance *prpC* and *prpD* which encode enzymes from the methylcitrate cycle are required for growth of *M. tuberculosis* on propionate medium or in murine macrophages but not in mice [32]. Likewise a recent study reports that the heavy metal efflux P-type ATPase CtpC is required for *in vitro* growth of *M. tuberculosis* in zinc-containing medium and in macrophages but is dispensable for multiplication during mouse infection [33]. Finally some enzymes involved in glycolysis are dispensable for *in vivo* growth and virulence of various *M. bovis* strains but are needed for their *in vitro* growth on glycerol-containing medium [34]. Therefore, essentiality *in vivo* is a crucial information for drug target validation that cannot be simply deduced from requirement of an enzyme for *in vitro* growth in defined medium.

Following PptT depletion, *M. tuberculosis* and *M. bovis* BCG failed to multiply *in vitro*, both on solid and liquid media, and *in vivo* in the spleens of SCID mice for *M. bovis* BCG and during the acute phase of infection in immunocompetent mice for *M. tuberculosis*. Presumably, growth stopped due to the interruption of *de novo* mycolate synthesis, which requires activation of the PKS13 enzyme by PptT [16]. Similarly, treatment of infected mice with drugs, such as isoniazid or ethionamide, that target mycolic acid biosynthesis severely impedes the growth of the infecting mycobacteria during the acute phase [35].

We also established that PptT deficiency impairs survival of *M. bovis* BCG in macrophages and in the lung of SCID mice and markedly reduces persistence of *M. tuberculosis* during the chronic phase of infection in mice. Interpretation of these results is complicated by the fact that the replicative and metabolic state of the bacteria during the persistent infection phase remains unclear. The current view of chronic tuberculosis is that bacteria are in a non-replicative state or have low level of replication [23], [36]. If this model is correct, the impaired survival of *M. tuberculosis* in mice was probably not solely due to disrupted mycolic acids biosynthesis because mycolic acid synthesis during the chronic phase is probably low. Indeed, antibiotics which affect the synthesis of mycolic acid have only poor bactericidal activity during the chronic phase of infection in mice [37]. Alternatively, the constant bacterial load in mice during persistence may be the consequence of a balance between active bacterial replication and killing by the host immune system [22], [23]. In this situation, PptT deficiency may abolish PKS13 activation and consequently synthesis of mycolic acids required for bacterial multiplication, resulting in the observed phenotype *in vivo*. This dynamic model may also explain why the *M. bovis* BCG mutant was eliminated from SCID mice in the absence of PptT.

The observed phenotypes of conditional *pptT* mutants may also be the consequence of additive effects. PptT activates not only PKS13 but also other type-I PKS and NRPS required for the formation of polyketide-derived lipids and mycobactins. Polyketide-derived lipids include phthiocerol dimycocerosates (DIM) and the structurally related phenolglycolipids (PGL), the trehalose ester families that include sulfolipids (SL), diacyltrehalose (DAT) and polyacyltrehalose (PAT) and the mannosyl-β-1-phosphomycoketides which are incorporated into the mycobacterial cell envelope [11], [12]. Several lines of evidence indicate that some of these lipids, such as DIM, contribute to cell envelope properties, such as the permeability barrier [38]. Therefore, the absence of these molecules may contribute to bacterial sensitivity to the bactericidal activities of phagocytes. In addition, some of the compounds requiring PptT for their biosynthesis exhibit other properties which might be important for the interaction with the host. For instance, DIM improve the invasiveness of *M. tuberculosis* and its capacity to arrest phagosome maturation, possibly by inducing changes in the plasma membranes of human macrophages during infection and several studies have reported that trehalose dimycolates (TDM) may regulate the host cell response [39]–[42]. The *in vivo* phenotype of our conditional mutants in mice may therefore be a consequence of the loss of various mycobacterial lipids that contribute to pathogenicity through modulation of the host immune response.

Finally, we cannot exclude the possibility that PptT contributes to persistence through its role in mycobactin siderophore biosynthesis. *M. tuberculosis* produces siderophores under iron-limiting conditions, particularly in infected hosts, and *M. tuberculosis* carrying mutations in genes involved in the synthesis or in the transport of siderophores are attenuated for survival in human macrophages and in the lungs of infected mice [43]–[45]. Although there is no experimental data formally demonstrating that inactivation of *pptT* results in mycobactin deficiency, PptT *in vitro* activates MbtB and MbtE, two NRPS involved in mycobactin assembly and is also expected to activate MbtF and MbtD both proteins also encoded by the mycobactin gene cluster [17].

Clearly, further experiments are necessary to determine whether the impaired survival of *M. tuberculosis* in the absence of PptT reflects the incapacity of the mutant to maintain its envelope integrity and to multiply, its failure to modulate the immune response or its reduced capacity to acquire iron. Conditional mutations of genes specifically involved in the production of each class of lipid, including mycolic acids, and of siderophores would be useful: testing the behavior of such mutant strains in the intracellular environment of macrophages and in the murine model would be informative.

In this study, we also present the development of a Scintillation Proximity Assay (SPA) for HTS to identify PptT inhibitors. Several assays amenable to HTS, including FRET-based assays and fluorescent polarization assays, have been reported for the discovery of PPTase inhibitors [25], [26], [46]. However, these approaches involve the use of CoA–fluorescent dye conjugates, and we found that they are not suitable for screening PptT inhibitors because PptT is unable to use modified CoA analogs as substrates. An assay using BpsA as a reporter for PPTase activity was recently described [47]. BpsA is an NRPS protein that catalyzes conversion of two L-glutamines into indigoidine, a blue pigment that can be detect by spectrometry; thus, PPTase activity can be assayed by measuring indigoidine synthesis following activation of BpsA by the PPTase. This assay is only applicable to PPTases that activate the PCP domain of BspA and furthermore inhibitors identified by this approach would include both PPTase inhibitors and BspA inhibitors.

Therefore, we developed an alternative approach based on the SPA technology. This involves the use of scintillating microspheres that emit light when a radiolabeled molecule is in close proximity. This approach has been successfully applied to a wide variety of enzyme assays and for various purposes including receptor binding assays, radioimmunoassays, and studies of protein-protein and protein-DNA interactions [27], [48]. We used [^3^H]CoA: CoA is the natural substrate of PptT, and ^3^H is ideally suited for SPA because it emits low energy beta particles such that non-proximity effects are minimized [48]. We optimized assay conditions with respect to cost, speed, sensitivity and robustness. We demonstrated that the assay was stable, as evaluated by the Z′ factor, tolerant to DMSO, and suitable for detection of PptT inhibitors. It requires no prior separation step and can be easily automated. It was developed for use in 96-well plates but could be easily adapted to 384-well microplates for use with robotic equipment. In addition, this assay is effective for identifying inhibitors of Sfp (data not shown) and could be easily adapted for other PPTases by using appropriate ACP domains.

In conclusion this study establishes that PptT is required for mycobacterial growth and persistence in immunodeficient and immunocompetent animal models and led to the development of a novel enzymatic assay suitable for HTS of PptT inhibitors. These various findings validate PptT as an attractive target for anti-tuberculosis compounds and provide a tool to search for such molecules.

## Materials and Methods

### Ethics statement

All animal experiments were performed in animal facilities that meet all legal requirements in France and by qualified personnel in such a way to minimize discomfort for the animals. All procedures including animal studies were conducted in strict accordance with French laws and regulations in compliance with the European community council directive 68/609/EEC guidelines and its implementation in France. All protocols were reviewed and approved by the Comité d'Ethique Midi-Pyrénées (reference MP/04/26/07/03).

### Bacterial strains, growth media and culture conditions

Plasmids were propagated at 37°C in *Escherichia coli* DH5α in LB broth or LB agar (Invitrogen, CergyPontoise, France) supplemented with either Km (40 µg/ml) or Hyg (200 µg/ml). ACP and ACPb domains were produced in *E. coli* BL21(DE3)Δ*entD*, a bacterial strain which contains a 533-nucleotide deletion within the *entD* gene [16]. *M. bovis* BCG and *M. tuberculosis* H37Rv wild-type strains and the PMM99 and PMM168 recombinant strains were grown at 37°C in Middlebrook 7H9 broth (Invitrogen) containing ADC (0.2% dextrose, 0.5%BSA fraction V, 0.0003% beef catalase) and 0.05% Tween-80 when necessary, and on solid Middlebrook 7H11 broth containing ADC and 0.005% oleic acid (OADC). Various concentrations of ATc (Sigma-Aldrich, St Louis, MO, USA) from 100 to 300 ng/ml were used to supplement 7H9 and 7H11 for PMM99 and PMM168 cultures. When required, Km, Hyg and Str were used at concentrations of 40 µg/ml, 50 µg/ml and 25 µg/ml, respectively.

### Construction of plasmids for the production of MBP-PptT, ACP and ACPb

pMSTB was constructed by amplifying the *pptT* gene of *M. tuberculosis* from genomic DNA with oligonucleotides tubSfp3 and tubSfp4 (Table 1). The PCR product was inserted into the XmnI site of pMAL-C2x (New England Biolabs, Evry, France). The resulting plasmid was digested with NdeI and BamHI and the 1.86 kb NdeI-BamHI fragment carrying the *pptT* gene fused to *malE* was ligated between the NdeI and BamHI sites of pET26b (Novagen, Madison, WI, USA) downstream from the T7 promoter to yield pMSTB. This vector allowed the production of PptT with MBP fused to its N-terminus. To generate pSC994, the *pks13* gene from *M. tuberculosis* was fragmented with DNAse-I and sized fragments (500–1500 bp) isolated by preparative agarose gel electrophoresis and ligated into a DHFR insertion vector. Fragments ligated in-frame were isolated based on survival of colonies on agar plates supplemented with 6 µg/ml trimethoprim [49]. This secondary library was subcloned into the split-GFP solubility vector for *in vivo* and *in vitro* solubility assays [50]. One DNA fragment was selected from eight candidates covering the N-terminal ACP domain of PKS13 and ligated between the NdeI/BamHI restriction sites of pET-N6his expression vector for large-scale protein production. This vector allowed the production of the ACP fragment of PKS13 (residues M1-V122) fused to a polyhistidine tag at N-terminus.

**Table 1:** Strains, plasmids and oligonucleotides used in this study.

Name	Relevant characteristics/sequence	Ref./source
*Strain*		
PMM99	*M. bovis* BCG Pasteur Δ*pptT::km* carrying pCpptTmb, Km^R^Hyg^R^	[16]
PMM168	*M. tuberculosis* H37Rv Δ*pptT::km* carrying pCpptTmb, Km^R^Hyg^R^	This study
*Plasmid*		
pCpptTmb	pSE100 containing *pptT* and the *p_imyc_-tetR* expression cassette, Hyg^R^	[16]
pSTB	pET26b containing *pptT* from *M. tuberculosis* H37Rv fused to a DNA fragment encoding a poly-histidine tag, Km^R^	This study
pMSTB	pET26b containing *pptT* from *M. tuberculosis* H37Rv fused to *malE*, Km^R^	This study
pSC994	pET28 containing DNA encoding the ACP fragment of PKS13 (residues M1-V122) from *M. tuberculosis* H37Rv, Km^R^	This study
pHGF8A26	pET28 containing DNA encoding the ACP fragment of PKS13 fused to an AviTag peptide sequence, Km^R^	This study
pMV361st	pMV361e harboring a streptomycin-resistance gene, Str^R^	This study
*Primer*		
res1	5′-GCTCTAGAGCAACCGTCCGAAATATTATAAA-3′	
res2	5′-TATCGGACAAGCAGTGTCTGTTA-3′	
pptTUP	5′-ACCCGTCGCCGAATCGCTG-3′	
pptTDW	5′-ACCCTCGTCACGCAGCAGC-3′	
pptT5	5′-GCTTGGCAACCGATCGTGCG-3′	
tubSfp3	5′-ATGACGGTAGGCACGCTGG-3′	
tubSfp4	5′-CTATAGCACGATCGCGGTC-3′	
ACPb3	5′-GATCCGGTGGCCTGAATGACATCTTTGAGGCCCAGAAGA TCGAGTGGCATGAGAACCTGTACTTCCAGGGATAAC-3′	
ACPb4	5′-TCGAGTTATCCCTGGAAGTACAGGTTCTCATGCCACTCG ATCTTCTGGGCCTCAAAGATGTCATTCAGGCCACCG-3′	

To generate vector pHGF8A26, synthetic AviTag-encoding DNA fragments (ACPb3 and ACPb4, Table 1) were annealed and inserted between the BamHI and XhoI restriction sites of pSC994 downstream from the DNA fragment encoding the ACP domain of PKS13. This vector was used to produce an ACP domain fused to a N-terminal (His)_6_tag for purification and a C-terminal AviTag (GGLNDIFEAQKIEWHENLYFQG) for enzymatic biotinylation (ACPb). pMV361st was constructed by insertion of the SphI-SphI DNA fragment conferring Str resistance from pGC76 into the SmaI restriction site of pMV361e, a pMV361 derivative containing the pblaF* promoter in place of the original *phsp60* promoter [51].

### Construction of the *M. tuberculosis* PMM168 mutant strain

To construct the PMM168 mutant of *M. tuberculosis*, we used the strategy described for the construction of the *M. bovis* BCG mutant PMM99 [16]. Complementation plasmid pCpptTmb which harbors the *pptT* gene (*Rv2794c*) from *M. tuberculosis* under the control of the *p_myc1_tetO* promoter and the *tet*R gene under the *p_imyc_* promoter [16], [18] (Figure S1) was transferred into *M. tuberculosis* H37Rv. Transformants were selected on 7H11 agar plates supplemented with OADC and Hyg. A derivative of the mycobacteriophage phAE87 that contains a disrupted *pptT::km* allele [16], [52] was used to disrupt the chromosomal copy of *pptT* in the resulting *M. tuberculosis*:pCpptTmb strain as previously described [52]: allelic exchange mutants were selected on 7H11 agar plates supplemented with OADC, Hyg, Km, and ATc at a concentration of 300 ng/ml at 37°C. Genomic DNA was extracted from several clones [53] and analyzed by PCR using various primers (Figure S1 and Table 1). One clone named PMM168 (Δ*pptT*:pCpptTmb) in which the wild-type copy of *pptT* has been replaced by the *pptT::km* allele was selected.

### Production and purification of MBP-PptT, ACP and ACPb

A starter culture of *E. coli* BL21(DE3) transformed with pMSTB was used to inoculate 50 ml of LB broth containing Km. The culture was grown at 37°C until the OD_600_ reached 0.4 and MBP-PptT expression was then induced by addition of 0.5 mM IPTG. The culture was continued for 3.5 hours at 24°C, and cells were harvested by centrifugation (4000 rpm, 15 min, 4°C). The cells were resuspended in 2.5 ml of 20 mM Tris.HCl pH7.0, 200 mM NaCl, 1 mM EDTA and lyzed by addition of 1 mg lysozyme. The extract was sonicated three times for 1 min (Vibracell, Bioblock scientific, Illkirch, France) and centrifuged (75000 g, 30 min, 4°C). The supernatant (about 2.5 ml) was loaded onto a 1 ml MBP Trap column (GE healthcare, Vélizy, France) previously equilibrated with 20 mM Tris.HCl pH7.0, 200 mM NaCl, 1 mM EDTA and washed with 3 ml of the same buffer. The MBP-PptT protein was eluted with 4×1 ml of 20 mM Tris.HCl pH7.0, 200 mM NaCl, 1 mM EDTA, 10 mM maltose. The most concentrated protein fraction (1 ml) was dialyzed overnight against 10 mM Tris.HCl pH8.0, 100 mM NaCl and stored at −80°C in 10% glycerol.

For the production of the ACP fragment from PKS13, a culture of *E. coli* BL21(DE3)Δ*entD* transformed with pSC994 was grown at 37°C until the OD_600_ reached 0.8. Expression was induced by addition of 1 mM IPTG and the culture was incubated for 3 h at 30°C. Bacteria were harvested by centrifugation (4000 rpm, 15 min, 4°C) and resuspended in 50 mM Tris.HCl pH8.0, 300 mM NaCl, 5 mM imidazole (6 ml). Cells were lyzed by addition of 2 mg of lysozyme and the extract was sonicated three times for 1 min. The soluble *E. coli* extract (6 ml) was recovered by centrifugation (20000 g, 15 min, 4°C) and loaded onto a HiTrap column (1 ml) (GE healthcare) previously equilibrated with 50 mM Tris.HCl pH8.0, 300 mM NaCl, 5 mM imidazole. The column was washed with 4 ml of 50 mM Tris.HCl pH8.0, 300 mM NaCl, 10 mM imidazole and the ACP fragment was eluted with 4×1 ml of 50 mM Tris.HCl pH8.0, 300 mM NaCl, 450 mM imidazole. The most concentrated elution fraction (1 ml) was dialyzed for 2 h against 20 mM Tris.HCl pH8.0, 50 mM NaCl and stored at −80°C in 20% glycerol. The ACPb domain was produced and purified by the same procedure except that the strain used was an *E. coli* BL21(DE3)Δ*entD* transformed with pHGF8A26. After purification, the ACPb fragment was biotinylated *in vitro* using a BirA500 kit (Avidity, Aurora, CO, USA). In a typical reaction, 100 µM of purified ACPb was mixed with 100 µl of biomix A, 100 µl of biomix B and 7.5 µl of BirA (0.28 µM) in a total volume of 1 ml and incubated at 30°C for 1 hour. Biotinylated ACPb was stored at −80°C in 20% glycerol. Biotinylation efficiency was checked by incubating 5 µl of biotinylated ACPb with 10 µl of streptavidin at 75 µM (Sigma-Aldrich) for 1 hour at room temperature followed by SDS-PAGE.

### Assays for *apo*-ACP to *holo*-ACP conversion with CoA and CoA analogs

CoA and acetyl-CoA were purchased from Sigma-Aldrich and CoA analogs (CoA488, CoA547 and CoA-biotin) were from New England Biolabs. Reactions were performed in Eppendorf tubes in a total volume of 50 µl at 30°C by incubating 200 nM of MBP-PptT with 10 µM of ACP and 10 µM of CoA or 10 µM of a CoA analog in the presence of 75 mM Tris.HCl pH 7.0, 10 mM MgCl_2_, 30 mM NaCl, and 25 mM DTT. For reactions in the presence of CoA or acetyl-CoA, production of the *holo*-ACP form was analyzed by loading 18 µl of reaction mix on a 12% polyacrylamide gel containing 2.5 M urea to separate the *apo*- and *holo*-ACP forms [54], [55]. Protein bands were revealed by Coomassie blue staining. When fluorescent CoA analogs (CoA547 or CoA488) were used, reaction products were separated by SDS-PAGE and fluorescence signals were detected with a GE Typhoon scanner (GE healthcare). Biotin-labeled ACP was detected by spotting 5 µl of the reaction mix onto a nitrocellulose membrane followed by streptavidin-peroxidase conjugate (Sigma-Aldrich) (1∶10000 in PBS containing 1% BSA for 1 h) and enhanced chemiluminescence detection. Sfp protein (200 nM; New England Biolabs) was used in control experiments.

### Scintillation Proximity Assay (SPA)

SPA assays were performed in white 96-well plates (PerkinElmer, Courtaboeuf, France) at 30°C. In a typical reaction, 80 nM of MBP-PptT was mixed with 4 µM of biotinylated ACPb, 2 µM of [^3^H]CoA (Biotrend, 185 Gbq/mmol, 20 µM) and 2 µM of CoA in a total volume of 10 µl containing 80 mM Tris.HCl pH 8.0, 20 mM MgCl_2_, 25 mM DTT. Negative controls involved leaving out PptT enzyme. Reactions were stopped by addition of 20 µl of stop buffer (200 mM EDTA) and 250 µg of streptavidin PVT SPA scintillation beads (PerkinElmer) resupended in 60 µl of water. Scintillation signals were detected using a TopCount (Perkin-Elmer). The Z′ factor was calculated thus: Z′ = 1−(3σ_c+_+3σ_c−_)/|μ_c+_−μ_c−_| where μ_c+_, μ_c−_, σ_c+_, σ_c−_ are the standard deviations (σ) and means (μ) of positive (+) and negative (−) control values [29].

### Growth curves of PMM99 and western blot analyses of crude cell lysates

A 5 ml culture of PMM99 grown in 7H9 medium supplemented with Tween-80 0.05% and ATc (100 ng/ml) was centrifuged (4000 rpm, 10 min, 25°C) and resuspended in 5 ml 7H9 medium supplemented with Tween-80. 200 µl were used to inoculate 200 ml of 7H9 medium supplemented with Tween-80 without or with different concentrations of ATc (0.1; 0.3; 1 and 100 ng/ml). In parallel 200 µl of a preculture of *M. bovis* BCG wild-type grown in 7H9 medium was used to inoculate 200 ml of 7H9 medium supplemented with Tween-80. Cultures were incubated at 37°C and bacterial growth rates were monitored by measuring the turbidity in cultures at 600 nm (OD_600_). After 6 days of culture, 10 ml of each culture were collected and further incubated at 37°C for OD measurements and 190 ml were used to prepare cell lysates. The cells were harvested by centrifugation (4000 rpm, 10 min, 4°C) and suspended in 500 µl of PBS buffer. Glass beads (in 500 µl) were added and cells were disrupted using a mini-bead beater for 3 min at RT. Cell debris was removed by centrifugation (10000 rpm, 10 min, 4°C) and the supernatants stored at −20°C for subsequent SDS-PAGE and western blot analyses. Protein concentrations in cell extracts were determined using the Bradford method.

Aliquots of protein extracts (0.5 to 5 µg) were incubated with sample buffer at 95°C for 10 min and subjected to SDS-PAGE. The gels were stained with Coomassie blue or the proteins were electrophoretically transferred to nitrocellulose membranes. The membranes were either incubated with an anti-PptT antibody (1/6250) or with an anti-Hsp65 antibody (1/2000000) (a generous gift from Dr. Daffé) and with an anti-rabbit peroxidase conjugate (1/20000; Biorad, Marnes-la-Coquette, France). Bound antibody was visualized by an enhanced chemiluminescence reaction (Millipore, Molsheim, France) and exposed to Fuji X- ray film.

### Testing the requirement for PptT *in vitro*

The wild-type strain of *M. tuberculosis* H37Rv was grown in 7H9 containing ADC and the PMM168 mutant on 7H9 containing ADC supplemented with Km, Hyg and ATc (100 ng/ml) for 7 days at 37°C. Dilutions of these cultures were streaked onto 7H11 plates supplemented with OADC with or without 300 ng/ml ATc. The plates were incubated for 20 days at 37°C. To show that PptT is required for the growth of *M. tuberculosis* in liquid medium, a 5 ml culture of PMM168 grown in 7H9 medium supplemented with Tween-80 and ATc (100 ng/ml) was centrifuged (4000 rpm, 10 min, 25°C) and resuspended in 5 ml 7H9 medium supplemented with Tween-80. 10 µl were used to inoculate flasks containing 10 ml of 7H9 supplemented with Tween-80, Km and Hyg with (3 flasks) or without (3 flasks) 100 ng/ml ATc. The cultures were incubated at 37°C and 100 µl of serial dilutions of the cultures on days 0, 4, 8 and 12 were streaked onto 7H11 plates supplemented with OADC with or without 300 ng/ml ATc. The plates were incubated for 20 days at 37°C. Similar experiments were performed with PMM99, the conditional *M. bovis* BCG mutant, except that an ATc concentration of 100 ng/ml was used in 7H11 plates.

### Macrophage infections

To prepare bone marrow-derived macrophages, femurs and tibiae were resected from 8-week old female BALB/c mice. Haematopoietic stem cells were collected by flushing the bone cavity with RPMI-1640 culture medium (Fischer Scientific, Illkirch, France). Erythrocytes were lysed by 2 min incubation in ACK buffer (Invitrogen, Saint Aubin, France) at room temperature. Cells were washed three times in HBSS and cultured for 6 days (with one refeed on day 3) in RPMI-1640 Glutamax I, 25 mM HEPES (Invitrogen) medium supplemented with 10% fetal calf serum, 1% sodium pyruvate, 1% non-essential amino-acids and 10% LADMAC conditioned medium containing colony stimulating factor-1 (M-CSF) for macrophage differentiation. LADMAC cells were cultured and conditioned medium produced as recommended by the ATCC (LGC Promochem, cell line number: CRL-2420). On day 6, cells were collected and both macrophage phenotype and purity (>90%) were analyzed for CD11b and F4–80 antigen expression with monoclonal antibodies anti CD11b (clone M1/70; BD Biosciences, Franklin lakes, NJ, USA) and anti F4–80 (clone CI:A3-1; AbDSerotec, Oxford, UK) using a FACScalibur flow cytometer (BD Biosciences). Bone marrow-derived macrophages were seeded at a density of 5×10^5^ cells in 24-well format plates and incubated at 37°C overnight to adhere to the plastic in complete RPMI without LADMAC conditioned medium. On day 7, cells were infected with PMM99 (the *M. bovis* BCG mutant) at a multiplicity of 10 bacteria per cell (10∶1); the bacterial inoculum was prepared as previously described [56]. Two hours later, the supernatant was discarded and the macrophages were thoroughly washed with HBSS to remove extracellular bacteria. They were then cultured in complete RPMI with or without 500 ng/ml ATc. After various times of incubation, cells were lysed by addition of 1 ml of 0.5% Triton X-100 in water to release intracellular bacteria. Aliquots of 100 µl of diluted cell lysates (1∶100) were plated on 7H11 agar containing Hyg, Km and ATc (100 ng/ml) to determine CFU.

### Animal experimentation

Female 7–8-week old SCID and BALB/cJ mice were purchased from Charles River Laboratory (L'arbresle, France) and Centre d'Elevage Janvier (Le Genest St Isle, France) respectively. To determine CFU counts in infected mice, bacteria were recovered from the spleen and lungs by homogenizing tissues in 5 ml of NaCl/Pi containing 0.05% Tween-80; 100 µl of serial dilutions of these lung or spleen homogenates were plated on 7H11 plates supplemented with the appropriate antibiotic(s). Colonies were counted, and the detection limit was 50 bacteria (1.7 log) per organ (100 µl of undiluted homogenate).

For the mixed-infection experiment in SCID mice, the *M. bovis* BCG wild-type strain was transformed with pMV361st, a mycobacterial plasmid conferring streptomycin resistance. Inocula were prepared by mixing *M. bovis* BCG:pMV361st cells with PMM99 cells at a final concentration of 1.23×10^8^ CFU/ml for the wild-type strain and 1.25×10^8^ CFU/ml for the PMM99 strain in a final volume of 2 ml. The actual numbers of CFU of each strain in the inocula were determined by plating serial dilutions on 7H11 plates containing Str and on 7H11 plates containing Km, Hyg and ATc (100 ng/ml) before infection. Twenty SCID mice were infected intranasally with 40 µl of the BCG:pMV361st/PMM99 mixture (4.92×10^6^ CFU of *M. bovis* BCG:pMV361H and 5×10^6^ CFU of PMM99). Five mice were killed one day after infection and used to determine the number of CFU that seeded in lungs; and five mice were killed on each 15, 30, 63 days after infection to count the number of bacteria in lungs and spleen. One mouse died before the end of the experiment. The numbers of viable bacteria in the organs of infected mice were determined by plating serial dilutions of tissue homogenates on 7H11 plates containing Str and on 7H11 plates containing Km and Hyg with or without 100 ng/ml ATc.

To establish toxicity of doxycycline for growing *M. bovis* BCG in SCID mice, 17 SCID mice were infected with the *M. bovis* BCG wild-type strain (4×10^5^ CFU/mouse). Three mice were killed one day post-infection to determine the number of CFU that seeded in lungs and the remaining mice were divided into four subgroups. Mice of subgroup 1 (n = 3) did not receive doxycycline; subgroups 2 (n = 4), 3 (n = 4) and 4 (n = 3) were provided with drinking water supplemented with 5% sucrose and doxycycline at concentrations of 0.1, 0.3 and 1 mg/ml, respectively. Mice were killed 21 days post-infection and CFU counts in lungs were determined by plating serial dilutions tissue homogenates on 7H11 plates.

For mice infected with PMM99, PMM99 was cultivated in 7H9 medium containing Hyg, Km and ATc (100 ng/ml). Nine SCID mice were infected intranasally with 40 µl of inoculum (4.79×10^5^ CFU/mouse). Three mice were killed one day post-infection for CFU counting. Three mice were provided with drinking water containing 5% sucrose and doxycycline (0.1 mg/ml) and three mice were maintained on normal drinking water. The mice were killed 21 days post-infection and CFU counts in lungs and spleen were determined by plating serial dilutions of tissue homogenates on 7H11 plates containing Km and Hyg in the presence or in the absence of ATc (100 ng/ml).

To study the involvement of PptT in the multiplication of *M. tuberculosis* during the acute phase of infection, 16 BALB/c mice were infected intranasally with *M. tuberculosis* H37Rv (1.72×10^3^ CFU/mouse) and 16 BALB/c mice were infected with PMM168 (0.44×10^3^ CFU/mouse). Three mice from each group were killed 1 day post-infection to determine bacterial uptake. The remaining 13 mice in each group were divided in two subgroups: seven mice were fed drinking water supplemented with 5% sucrose and doxycycline (0.1 mg/ml) and the other six mice did not receive doxycycline. Three mice of the doxycycline-treated subgroup and three mice of the non-treated subgroup were killed on day 14 and 4 mice of the treated subgroup and 3 mice of the no-treated subgroup were killed on day 28; CFU counts in lungs and spleen were determined by plating serial dilutions of lung and spleen homogenates. 7H11 plates were used for mice that were infected with the wild-type strain of *M. tuberculosis* and 7H11 plates containing Km and Hyg with and without ATc (300 ng/ml) were used for mice that were infected with PMM168.

To establish the role played by PptT in *M. tuberculosis* persistence in mice, 78 mice were infected intranasally with 40 µl (2.65×10^2^ CFU/mouse) of the PMM168 mutant. Nine mice were killed 1 day after infection to determine bacterial loads in lungs and spleen. The remaining mice (69) were maintained on drinking water with doxycycline (0.1 mg/ml) for 35 days to induce expression of *pptT* in the mutant strain. On day 35, eleven mice were sacrificed to evaluate the multiplication of the bacteria in mice; doxycycline treatment was withdrawn from 29 mice and continued for 29 mice. Six mice of each subgroup were killed on days 63, 91 and 120 and the remaining mice (22) were killed on day 160. CFU counts in lungs and spleens of all mice were determined by plating serial dilutions of organ homogenates on 7H11 plates containing Km and Hyg in the presence or the absence of ATc (300 ng/ml).

## Supporting Information

Figure S1 **Construction and characterization of the** ***M. tuberculosis*** **mutant strain PMM168.** **A.** Representation of complementation plasmid pCpptTmb. **B.** Schematic diagram of the genomic organization of the *pptT* locus in wild-type *M. tuberculosis* and strain PMM168 (Δ*pptT*:pCpptTmb). Black, hatched, and white boxes on the chromosome represent the *pptT* gene, the fragment deleted during the construction of the knockout mutant, and the 5′ and 3′ flanking regions amplified by PCR for mutant construction, respectively. The *km*-resistance cassette used for targeted disruption is represented by a gray box. **C.** Strain PMM168 was analyzed by PCR, using various combinations of specific primers, as indicated. Positions and names of primers are indicated by arrows above each genetic structure, and the expected sizes of the PCR products are indicated.
(PDF)

Figure S2 **PMM99 crude cell lysates analyses.** Crude cell lysates of PMM99 (5 µg/lane) cultivated for 6 days in the presence of ATc (100, 1, 0.3 ng/ml) and a crude cell lysate of the *M. bovis* BCG wild-type strain (5 µg) were analyzed by SDS-PAGE and Coomassie blue staining (**A**) and by western blotting with an anti-Hsp65 antibody (**B**). M: PageRuler prestained protein ladder plus (Fermentas).
(PDF)

Figure S3 **Effect of doxycycline treatment on the survival of** ***M. bovis*** **BCG in SCID mice.** SCID mice were infected with wild-type *M. bovis* BCG and received 0.1, 0.3 or 1 mg/ml or no doxycycline in drinking water from the following day. Numbers of CFU in the lungs of infected mice on day 1 (D1) and in the lungs of untreated (dark gray bar) and doxycycline-treated (light gray bars) mice on day 21 (D21) were determined by plating dilutions of homogenized tissue on 7H11 media. Numbers on light gray bars indicate the concentration of doxycycline used. Values are means ± standard deviations (error bars) of CFU counts for three mice.
(PDF)

Figure S4 **Effect of pH and temperature on PptT activity.** PptT (200 nM) was incubated for 3 hours with the *apo*-ACP module (10 µM) and CoA (10 µM) in various buffers of different pH containing 10 mM MgCl_2_, 30 mM NaCl, and 25 mM DTT at 30°C (upper panel) or in the presence of 75 mM Tris.HCl pH 7.0, 10 mM MgCl_2_, 30 mM NaCl, and 25 mM DTT at 25, 30 or 37°C (lower panel). *apo*- (a) and *holo*-ACP (h) forms were separated on urea polyacrylamide gels and revealed by Coomassie blue staining.
(PDF)

Figure S5 **Effect of DMSO on SPA assay signal.** SPA assays were done in standard conditions in the absence or in the presence of 1–5% (vol/vol) DMSO. Reactions were stopped after 1 hour and scintillation signals were detected using a TopCount. Data are expressed relative to the mean of signals obtained in the absence of DMSO. Data are from one experiment performed in triplicate.
(PDF)

Protocol S1 **Preparation of anti-PptT polyclonal antibodies.**
(PDF)
