# Supplementary figures and images for: 4′-Phosphopantetheinyl Transferase PptT, a New Drug Target Required for Mycobacterium tuberculosis Growth and Persistence In Vivo
Source: PLoS Pathog. 2012 Dec 20;8(12):e1003097. doi: 10.1371/journal.ppat.1003097 (PMC3534377; doi:10.1371/journal.ppat.1003097)

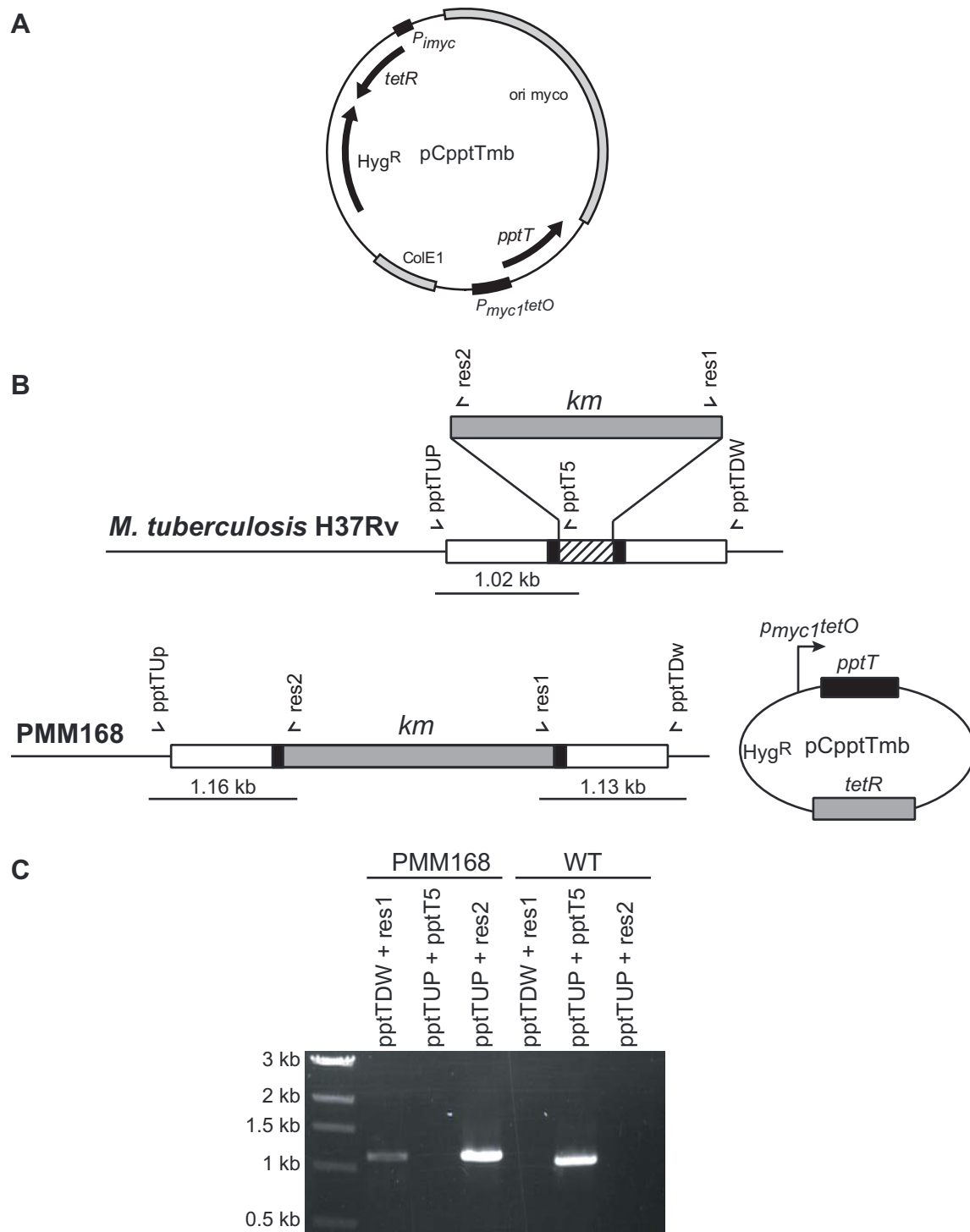

Figure S1

Supplement: Figure S1 — Construction and characterization of the M. tuberculosis mutant strain PMM168. A. Representation of complementation plasmid pCpptTmb. B. Schematic diagram of the genomic organization of the pptT locus in wild-type M. tuberculosis and strain PMM168 (ΔpptT:pCpptTmb). Black, hatched, and white boxes on the chromosome represent the pptT gene, the fragment deleted during the construction of the knockout mutant, and the 5′ and 3′ flanking regions amplified by PCR for mutant construction, respectively. The km-resistance cassette used for targeted disruption is represented by a gray box. C. Strain PMM168 was analyzed by PCR, using various combinations of specific primers, as indicated. Positions and names of primers are indicated by arrows above each genetic structure, and the expected sizes of the PCR products are indicated. (PDF) [file ppat.1003097.s001.pdf]

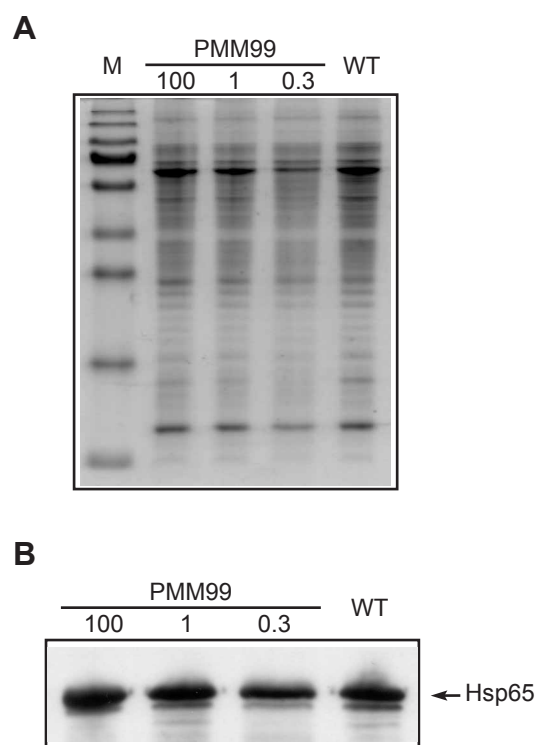

Figure S2

Supplement: Figure S2 — PMM99 crude cell lysates analyses. Crude cell lysates of PMM99 (5 µg/lane) cultivated for 6 days in the presence of ATc (100, 1, 0.3 ng/ml) and a crude cell lysate of the M. bovis BCG wild-type strain (5 µg) were analyzed by SDS-PAGE and Coomassie blue staining (A) and by western blotting with an anti-Hsp65 antibody (B). M: PageRuler prestained protein ladder plus (Fermentas). (PDF) [file ppat.1003097.s002.pdf]

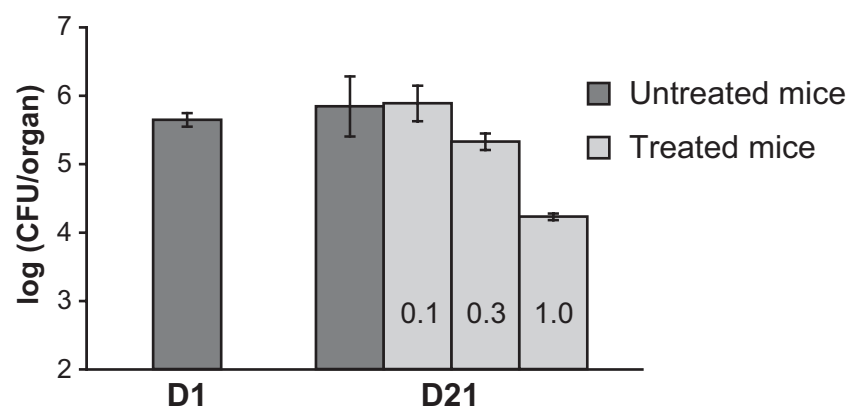

Figure S3

Supplement: Figure S3 — Effect of doxycycline treatment on the survival of M. bovis BCG in SCID mice. SCID mice were infected with wild-type M. bovis BCG and received 0.1, 0.3 or 1 mg/ml or no doxycycline in drinking water from the following day. Numbers of CFU in the lungs of infected mice on day 1 (D1) and in the lungs of untreated (dark gray bar) and doxycycline-treated (light gray bars) mice on day 21 (D21) were determined by plating dilutions of homogenized tissue on 7H11 media. Numbers on light gray bars indicate the concentration of doxycycline used. Values are means ± standard deviations (error bars) of CFU counts for three mice. (PDF) [file ppat.1003097.s003.pdf]

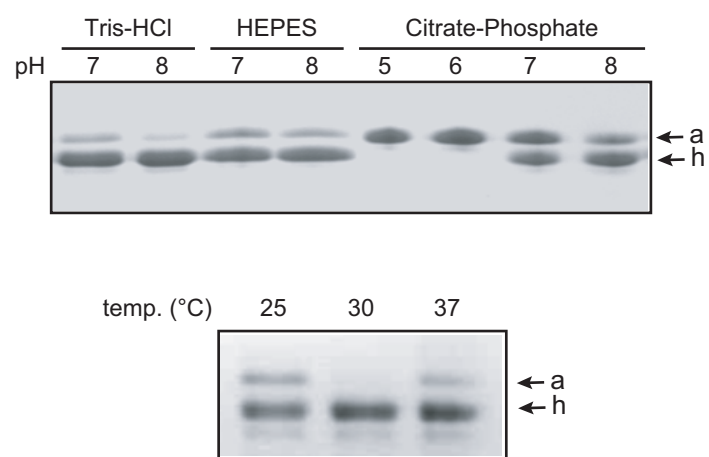

Figure S4

Supplement: Figure S4 — Effect of pH and temperature on PptT activity. PptT (200 nM) was incubated for 3 hours with the apo-ACP module (10 µM) and CoA (10 µM) in various buffers of different pH containing 10 mM MgCl2, 30 mM NaCl, and 25 mM DTT at 30°C (upper panel) or in the presence of 75 mM Tris.HCl pH 7.0, 10 mM MgCl2, 30 mM NaCl, and 25 mM DTT at 25, 30 or 37°C (lower panel). apo- (a) and holo-ACP (h) forms were separated on urea polyacrylamide gels and revealed by Coomassie blue staining. (PDF) [file ppat.1003097.s004.pdf]

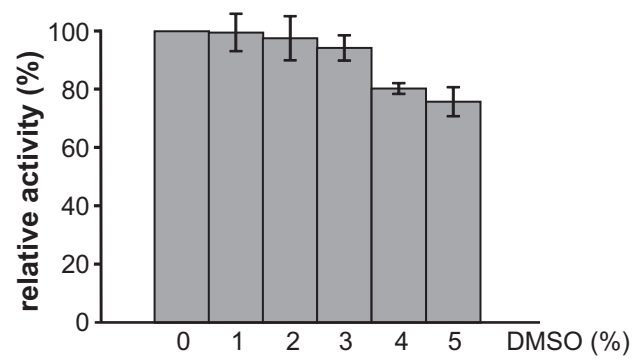

Figure S5

Supplement: Figure S5 — Effect of DMSO on SPA assay signal. SPA assays were done in standard conditions in the absence or in the presence of 1–5% (vol/vol) DMSO. Reactions were stopped after 1 hour and scintillation signals were detected using a TopCount. Data are expressed relative to the mean of signals obtained in the absence of DMSO. Data are from one experiment performed in triplicate. (PDF) [file ppat.1003097.s005.pdf]
