# Supplementary material for: 4′-Phosphopantetheinyl Transferase PptT, a New Drug Target Required for Mycobacterium tuberculosis Growth and Persistence In Vivo
Source: PLoS Pathog. 2012 Dec 20;8(12):e1003097. doi: 10.1371/journal.ppat.1003097 (PMC3534377; doi:10.1371/journal.ppat.1003097)
Supplement: Protocol S1 — Preparation of anti-PptT polyclonal antibodies. (PDF) [file ppat.1003097.s006.pdf]

Protocol S1

<u>Preparation of anti-PptT polyclonal antibodies</u>

Anti-PptT antibodies were obtained by immunization of two rabbits with the PptT protein from *M. tuberculosis*. Briefly, the *pptT* gene was amplified by PCR from *M. tuberculosis* H37Rv genomic DNA and inserted into pET26b (Novagen), downstream from the T7 promoter to yield plasmid pSTB. This encodes a recombinant PptT protein fused to a poly-His tag at its C-terminus. The construct was overexpressed in *E. coli* BL21(DE3) by incubation at 37°C for 3 hours with 0.4 mM IPTG. Bacterial cells were harvested and inclusion bodies, containing the PptT protein, were recovered and resuspended in binding buffer containing 6M urea (80 mM Tris-HCl pH7.9, 500 mM NaCl, 5 mM imidazole, 6M urea). PptT was purified on Ni-NTA His-Bind resin (Novagen) under denaturing conditions with 6M urea as described by the manufacturer. Aliquots were prepared by diluting 200 µg of purified PptT in a final volume of 400 µl with PBS buffer and stored at -80°C. Every 15 days, two aliquots were thawed, mixed 1:1 with Freund's incomplete adjuvant (Sigma-Aldrich) and injected into two rabbits. One week after each injection, 2 ml of serum were taken from the rabbits and anti-PptT antibody was titered. After five injections, rabbits were killed and total sera were recovered, incubated at 37°C for 1 h and at 4°C for one night and centrifuged (5000 rpm, 4°C, 10 min). The supernatant was stored at -80°C.
